# Recombinant Forms of α-Amylase AmyBL159 from a Thermophilic Bacterium *Bacillus licheniformis* MGMM159: The Effect of the Expression System on the Enzyme Properties

**DOI:** 10.3390/microorganisms13122747

**Published:** 2025-12-02

**Authors:** Elvira R. Suleimanova, Elizaveta A. Klochkova, Shamil Z. Validov, Marina P. Kolomytseva, Alexey M. Chernykh, Natalia V. Trachtmann

**Affiliations:** 1 Federal Research Center, Kazan Scientific Center of Russian Academy of Science, ul. Lobachevskogo, 2/31, Kazan 420111, Tatarstan Republic, Russia; elvira.suleymanova.20@mail.ru (E.R.S.); lisa.klochkova@gmail.com (E.A.K.); n.trahtman@knc.ru (N.V.T.); 2 Federal Research Center, Pushchino Scientific Center for Biological Research of the Russian Academy of Sciences, G.K. Skryabin Institute of Biochemistry and Physiology of Microorganisms, Prosp. Nauki 5, Pushchino 142290, Moscow Region, Russia; mkolomytseva@rambler.ru (M.P.K.); achernykh@rambler.ru (A.M.C.)

**Keywords:** α-amylase, thermostability, heterologous expression, *Bacillus licheniformis*

## Abstract

We present the cloning and heterologous expression of the α-amylase gene *amy*BL159 from a thermophilic strain *Bacillus licheniformis* MGMM159, which was isolated from wastewater sediments self-heated to 70 °C. The gene was successfully cloned into the pET22b and pHT01 vectors, expressed and AmyBL159_Ec_ and AmyBL159_Bs_ recombinant α-amylases were purified from *Escherichia coli* BL21(DE3)pLys and *Bacillus subtilis* 168 strains, respectively. The AmyBL159_Ec_ enzyme was most active in the range of 75–95 °C, while AmyBL159_Bs_ showed maximum activity at temperatures from 45 to 75 °C. AmyBL159_Bs_ was shown to be more thermostable. Both enzymes were active over a broad pH range of 4.0–12.0. It was shown that Mn^2+^ ions enhanced the activity of both enzymes (up to 163% for AmyBL159_Ec_ and 142% for AmyBL159_Bs_). These results highlight the importance of choosing an expression system for modulating the functional characteristics of recombinant α-amylase. The obtained AmyBL159_Ec_ and AmyBL159_Bs_ enzymes are promising for biotechnological applications under extreme conditions. The structure of the α-amylase was generated using the AlphaFold 3 web service. A structure–function analysis of this enzyme and previously studied α-amylases from *B. licheniformis* identified significant amino acid substitutions at positions 134(133) and 210(209) of the amino acid chain which may contribute to enhanced enzyme thermostability.

## 1. Introduction

Alpha-amylases (α-1,4-glucanohydrolase; EC 3.2.1.1) are a type of glycoside hydrolase that belong to the GH-13 family. The 1,4-alpha-D-glucanoglucanohydrolases can randomly cleave α-1,4 bonds between glucose molecules in both branched and unbranched forms of starch. The products of this reaction are maltose and maltotriose, which can be further converted by maltases into glucose [1,2,3]. Alpha-amylases are predominantly extracellular endoglycan hydrolases; however, in a number of microorganisms, the intracellular forms of these enzymes also perform important functions involved in cell growth processes [4,5]. Currently, microbial α-amylases have almost completely replaced chemical hydrolysis in industry, accounting for about 25% of the global enzyme market [6,7]. They are widely used in the food industry for the production of syrups and baking, in the textile industry to remove starch additives, and in the paper industry to modify cellulose. They are also used in fermentation processes, such as bioethanol production [8]. Nevertheless, although considerable progress has been made in studying and commercializing these enzymes, the existing range of α-amylases falls short of fully addressing the escalating needs of various industries. This is especially true for the demand for highly thermostable enzymes required in high-temperature processes [9,10,11], and for those resistant to extreme pH values, a critical property for use in detergents or in acidic hydrolysis conditions within the food industry [12,13,14,15]. Among the microorganisms that produce α-amylase, a special place is taken by bacteria of the genus *Bacillus*, including species such as *B. subtilis*, *B. stearothermophilus*, *B. licheniformis*, and *B. amyloliquefaciens*. These bacteria have high secretory activity and are well-adapted to industrial conditions, making them widely used in the commercial production of enzymes [3]. Obtaining α-amylases in an intracellular form provides a high level of expression, but it requires complex and expensive purification processes, including cell lysis (using ultrasound, specific enzymes, or chemical agents), followed by isolation of enzymes from the cell lysate. In contrast, secreting the enzyme into the culture medium significantly simplifies the process, as purification can be carried out directly from the culture fluid, eliminating the need for cell disruption and reducing the number of purification steps [16,17]. Gram-positive bacteria, such as *B. subtilis* and *B. licheniformis*, Gram-negative bacteria, like *Escherichia coli*, and actinobacteria, like *Corynebacterium glutamicum*, are widely used in the production of secretory α-amylases. These microorganisms are highly productive and easy to culture [18]. *B. subtilis* is a Gram-positive bacterium that is widely used in biotechnology for its ability to produce secretory enzymes. This microorganism has been granted the GRAS (Generally Recognized As Safe) status by the European Food Safety Authority (EFSA) [19], making it a preferred platform for food and feed enzyme production. The ability of *Bacillus* species to produce and secrete large amounts of extracellular enzymes, up to 25 g per liter has made them important in industrial fermentation processes [20,21,22]. Bacteria from the *Bacillus* genus have a key advantage in their ability to grow and ferment at high temperatures and in a wide range of pH conditions [23,24]. The use of such strains has made it possible to synthesize commercial enzymes with specified parameters: thermal stability, activity under extreme pH conditions, and optimal temperature activity for use in various industrial applications [20]. In addition, the production of secreted proteins in the bacterial system can provide better conditions for protein folding compared to reducing medium in the cytoplasm, thus preventing the formation of inclusion bodies [22].

This study describes the cloning and heterologous expression of the α-amylase gene (*amy*BL159) from the thermophilic strain *B. licheniformis* MGMM159 in two widely used microbial hosts: *Escherichia coli* and *Bacillus subtilis*. We present a comparative analysis of the biochemical properties of the resulting recombinant enzymes—AmyBL159_Ec_ (from *E. coli*) and AmyBL159_Bs_ (from *B. subtilis*)—with particular emphasis on their catalytic efficiency, thermostability, and pH tolerance. This systematic comparison provides insights into how the expression host influences enzyme characteristics, thereby facilitating the rational selection of production systems for industrial enzyme manufacturing.

## 2. Materials and Methods

### 2.1. Isolation and Identification of Bacterial Strain

The *Bacillus* spp. MGMM159 strain was isolated from sewage sludge, which spontaneously heats up to 70 °C during composting. The microorganisms isolated from the compost were tested for their amylolytic activity using dishes containing starch nutrient agar, with the following ingredients in g/L: soluble starch—5, peptone—5, yeast extract—5, MgSO_4_x 7H_2_O—0.5, FeSO_4_x 7H_2_O—0.01, NaCl—0.01 and agar—20. The cells were incubated at 37 °C for 48 h. After incubation, the cells grown on the plates were treated with 2.5% Lugol’s iodine solution, and their amylolytic activity was assessed by observing clear zones around the bacterial colonies on a dark blue background, due to the formation of an iodine–starch complex.

Strain identification was performed by 16S rRNA gene sequencing. Total DNA was isolated from the strain with the TRIzol kit (Invitrogen, Carlsbad, CA, USA) following the manufacturer’s protocol. Amplification of the full-length 16S rRNA gene fragment was carried out with universal primers 27fm (5′-AGAGTTTGATCMTGGCTCAG-3′) и 1522R (5′-AAGGAGGTGATCCAGCCGCA-3′) [25]. The amplified fragment was isolated from a 1% TAE agarose gel using a DNA purification kit (Evrogen, Moscow, Russia). The nucleotide sequence of the purified DNA fragment was determined using Sanger sequencing (Evrogen, Moscow, Russia). The sequences were assembled using the Clone Manager 9.0 software (USA) and analyzed using the NCBI BLAST web interface (https://blast.ncbi.nlm.nih.gov, accessed on 15 March 2024). The strain was identified based on the highest sequence similarity to strains of known species from the GenBank database.

### 2.2. Bacterial Strains, Vectors and Recombinant Plasmids

All strains and plasmids used and constructed in this work are present in Table 1.

### 2.3. Creating of Recombinant Plasmids

Using the complete genome sequence of *B. licheniformis* ATCC 14580 (GenBank: GCA_034478925.1) as a reference and leveraging the high nucleotide sequence homology of α-amylase genes from closely related microorganisms available in the NCBI database, specific primers were designed to amplify the α-amylase gene from the MGMM159 strain. For cloning the α-amylase gene into the pET22b expression vector (using the *Nde*I and *Xho*I restriction sites), the following primers were synthesized: amy-BL-NdeI 5′-TTTT**CATATG**AAACAACAAAAACGGCTTTACGCCC-3′ and amy-BL-XhoI-stop 5′-TTTT**CTCGAG**TCTTTGAACATAAATTGAAACCGACCC-3′ (restriction sites are indicated in bold). To clone the *amy*BL159 gene into the pHT01 vector for expression in *Bacillus subtilis* 168 and for its subsequent purification by affinity chromatography, a sequence encoding a 6xHis-tag was incorporated into the primer complementary to the 3′-end of the gene: amyBl-6his: 5′-GGGTCA*GTGGTGGTGGTGGTGGTG*CTCTCTTTGAACATAAATTGAAACCGAC-3′ (the 6xHis-tag sequence is shown in cursive). The restriction site *Bam*HI was introduced into the primer complementary to the 5′-end of the gene: amy-BL-BamHI: 5′-TTTT**GGATCC**ATGAAACAACAAAAACGGCTTTAC-3′ (the restriction site is indicated in bold). DNA fragments contained the *amy*BL159 gene were amplified using the high-fidelity PhantaMax DNA polymerase (Vazyme, Nanjing, China). Genomic DNA isolated from the *B. licheniformis* MGMM159 strain was used as the template for PCR. The PCR was performed under the following conditions: initial denaturation at 95 °C for 4 min (1 cycle); 30 cycles of denaturation at 95 °C for 45 s, primer annealing at 54 °C for 20 s, and elongation at 72 °C for 1 min 30 s; final extension at 72 °C for 2 min (1 cycle); and a final hold at 4 °C. The PCR products were purified from a 0.8% agarose gel (TAE buffer) using a DNA extraction kit (Evrogen, Moscow, Russia). The purified fragments were digested with the appropriate restriction enzymes and ligated into the pET22b vector (digested with *Nde*I and *Xho*I, for removing of the *pel*B sequence) to create the pET22b-amyBL159-6His construct, and into the pHT01 vector (digested with *Bam*HI and *Sma*I) to create the pHT01-amyBL159-6His construct. The DNA digestion and ligation reactions were carried out according to the manufacturer’s recommendations (SibEnzyme, Novosibirsk, Russia). The ligation mixture was used for transformation of *E. coli* DH5α cells using a previously described method [29]. Clones were selected on plates with LB medium supplemented with 100 µg/mL ampicillin. Plasmid DNA was isolated from positive clones using a commercial kit (Evrogen, Moscow, Russia). Isolated plasmids were verified by restriction analysis and sequencing (Evrogen, Moscow, Russia).

### 2.4. Heterologous Expression of the α-Amylase Gene in Cells of E. coli and B. subtillis

Cells of *E. coli* strain BL21(DE3)pLys and *B. subtilis* 168 were transformed with the resulting recombinant plasmids, pET22b-amyBL159-6His and pHT01-amyBL159-6His, respectively. The transformation of the *B. subtilis* 168 cells were carried out using a chemical transformation method, following a previously described protocols [30,31]. Following transformation, cells were selected on the dishes with LB medium supplemented with the ampicillin (100 µg/mL) and chloramphenicol (25 µg/mL) for *E. coli* BL21(DE3)pLys, and chloramphenicol (10 µg/mL) for *B. subtilis* 168. The resulting recombinant cells were inoculated into 50 mL of liquid LB medium containing the respective antibiotics and cultured overnight at 37 °C with shaking at 180 rpm. The overnight culture was diluted 1:50 with fresh LB medium supplemented with the appropriate antibiotics and incubated at 37 °C with shaking at 180 rpm until the optical density reached OD_600_ ≈ 0.6 for *E. coli* and OD_600_ ≈ 0.8 for *B. subtilis*. Expression of the *amy*BL159 gene was induced by adding isopropyl-β-D-thiogalactopyranoside (IPTG) to a final concentration of 0.5 mM, followed by incubation for 18 h at 18 °C with shaking (180 rpm) for *E. coli* and at 30 °C for *B. subtilis*.

### 2.5. Purification of the Recombinant Protein from the Culture Supernatant

The recombinant α-amylase AmyBL159, fused with 6xHis-tag at C-terminal, was purified from the culture supernatant of *E. coli* BL21(DE3)pLys (AmyBL159_Ec_) and *B. subtilis* 168 (AmyBL159Bs) using Ni-NTA affinity chromatography at 4 °C. Following expression, cells were centrifuged at 5000× *g* for 10 min at 4 °C, and the supernatant was loaded onto a Ni-NTA column pre-equilibrated with a buffer containing 50 mM Tris-HCl (pH 7.2), 300 mM NaCl, and 10 mM imidazole. Non-specifically bound proteins were removed by washing with the same buffer. The target enzyme was eluted using an elution buffer (50 mM Tris-HCl, pH 7.2, 200 mM NaCl, 300 mM imidazole, 3% glycerol). The protein concentration in the collected elution fractions was analyzed spectrophotometrically at 280 nm using a NanoDrop (Thermo Fisher Scientific, Waltham, MA, USA). Fractions with the highest protein content were pooled and concentrated using Vivaspin 6 ultrafiltration columns (Sartorius, Göttingen, Germany) with a 30 kDa molecular weight cut-off at 5000× *g* and 4 °C. The purity of the isolated protein was analyzed by denaturing electrophoresis on a 12% SDS-PAGE gel containing 25 mM Tris-HCl, 192 mM glycine, and 0.1% SDS (pH 8.3). Protein bands were visualized by staining the gel with a solution of 2% Coomassie Brilliant Blue R-250 solved in 20% ethanol, 10% acetic acid and 70% of water.

### 2.6. Activity Assay of α-Amylase

α-Amylase activity was determined by measuring the amount of reducing sugars released, using a previously described 3,5-dinitrosalicylic acid (DNS) method with minor modifications [32]. A 0.2% starch solution was used as the substrate for the amylase activity assay. The reaction mixture contained 0.2 µg of the purified α-amylase preparation in a total volume of 100 µL. The reaction was carried out at 55 °C in 50 mM sodium acetate buffer (pH 5.0) for 10 min. After incubation, the reaction mixture was combined with an equal volume of DNS reagent (44 mM 3,5-dinitrosalicylic acid, 1.06 M potassium sodium tartrate, 200 mM NaOH), boiled for 5 min, and cooled on ice for 5 min. Subsequently, 40 µL of the mixture was added to 160 µL of deionized water, and the absorbance was measured at 540 nm. The OD_540_ values of the no-enzyme control samples were subtracted from the values obtained after the 10-min incubation under the conditions described above. The concentration of sugars produced in each reaction mixture was determined using a glucose standard curve. All experiments were performed in triplicate, and the mean values and standard deviations were calculated. One unit of enzyme activity (U) was defined as the amount of enzyme that catalyzes the formation of 1 μmol of product per minute under optimal conditions. Specific activity was expressed as the amount of product formed (μmol) per milligram of enzyme per minute (U/mg).

### 2.7. Determining the Effect of Temperature, pH, Metal Ions, and Inhibitors on Amylase Activity

For all assays, the reaction was initiated by adding the recombinant enzyme to the reaction mixture at a final concentration of 2 µg/mL. α-Amylase activity was determined by measuring the amount of reducing sugars released using the 3,5-dinitrosalicylic acid (DNS) method described above. To determine the temperature optimum of the enzyme, the enzymatic reaction was carried out for 10 min at temperatures ranging from 45 to 95 °C in 10 °C increments. The assay was performed in 50 mM acetate buffer (pH 5.0) using a 0.2% soluble starch solution as the substrate. The pH dependence of enzyme activity was investigated at 55 °C using buffer systems covering a pH range of 4.0 to 12.0: 50 mM sodium acetate buffer (pH 4.0–6.0), 50 mM Tris-HCl buffer (pH 7.0–9.0), and 50 mM glycine-NaOH buffer (pH 10.0–12.0). The influence of metal cations on α-amylase activity was assessed by adding KCl, NaCl, CaCl_2_, MgSO_4_ and MnSO_4_ salts to a final concentration of 5 mM. The effect of inhibitors was evaluated by adding SDS and EDTA to final concentrations of 1% and 1 mM, respectively. Absorbance values were corrected by subtracting the blank (the reaction mixture without the enzyme, containing the corresponding salt).

### 2.8. Determination of Amylase Thermostability

The enzyme’s thermostability was determined by pre-incubating the purified enzyme at a concentration of 200 µg/mL at 60, 70, and 80 °C for various time intervals, followed by measuring the remaining enzymatic activity under optimal conditions (pH 5.0, 55 °C, 10 min). The reaction was initiated by adding the pre-treated enzyme to the reaction mixture at a final concentration of 2 µg/mL. Enzyme activity was determined using the DNS method described above. All experiments were performed in triplicate, and the mean values along with the standard deviations were calculated.

### 2.9. Obtaining a 3D-Model of the α-Amylase from B. licheniformis MGMM159 and Its Structural Analysis

The 3D-model of the α-amylase structure from *B. licheniformis* MGMM159 was obtained using the AlphaFold 3 program via the AlphaFold Server web service (https://alphafoldserver.com/welcome, accessed on 15 March 2024) [33]. The amino acid sequence of the α-amylase without the signal peptide and histidine tag in FASTA format was used as an input parameter. The quality of the obtained models was assessed using the SAVES v6.0 web server (https://saves.mbi.ucla.edu/, accessed on 15 March 2024). Visualization and analysis of the obtained models were performed in PyMol 2.5.0 [34].

### 2.10. Statistical Analysis

All experiments were performed in three biological replicates (n = 3) for each temperature. Data are presented as the mean ± standard deviation (SD). Statistical analyses were performed using GraphPad Prism version 8.0 (GraphPad Software, San Diego, CA, USA). Nonparametric comparisons between two groups were conducted using the Wilcoxon test (two-tailed). For multiple pairwise comparisons, *p*-values were adjusted using the Šidák correction to control the family-wise error rate. A two-sided *p* < 0.05 was considered statistically significant.

## 3. Results

### 3.1. Bacterial Strain Identification

**Figure 1:**
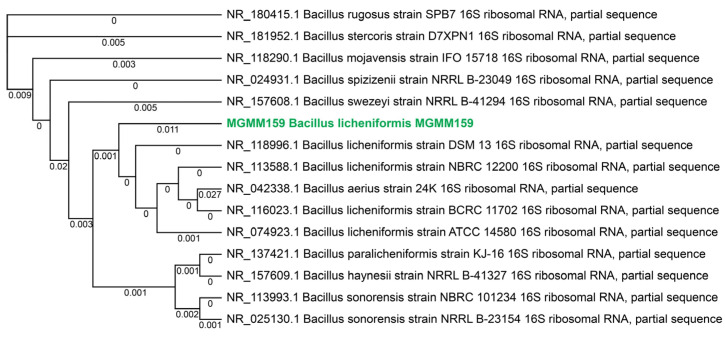
A phylogenetic tree of 16S rRNA sequences based on the results of a NCBI BLAST web interface (https://blast.ncbi.nlm.nih.gov, accessed on 15 March 2024)., multiple alignment using Clustal Omega version 1.2.3 (https://www.ebi.ac.uk/jdispatcher/, accessed on 15 March 2024), and reconstruction using the maximum likelihood method in PhyML algorithm Version 3.3.20220408 [35]. DNA sequences of 14 reference strains obtained from GenBank were included in the tree using the neighbor-joining method. The sequence *B. licheniformis* MGMM159 is highlighted in green.

The *Bacillus* spp. isolate MGMM159 was obtained from wastewater sediment that spontaneously heated up to 70 °C during the composting process (Kazan, Republic of Tatarstan, Russian Federation). The strain was selected on a nutrient agar plate containing starch to screen for α-amylase activity. After incubation and staining of the plates with an iodine solution, clear zones 4–5 mm in diameter formed around the colonies (Figure S1), indicating starch hydrolysis and confirming the presence of amylolytic activity in the bacterium. From the selected colony genomic DNA was extracted, and taxonomic identification was performed using 16S rRNA gene sequencing and analyzing. Based on the high sequence identity (99.8%) of the conserved region of the 16S rRNA gene, the *Bacillus* spp. isolate MGMM159 was identified as *B. licheniformis*. A phylogenetic tree of the 16S rRNA gene sequences is presented in Figure 1.

### 3.2. Cloning of the α-Amylase Gene from B. licheniformis MGMM159

A comparative analysis of α-amylase amino acid sequences from closely related strains was performed, revealing a very high degree of homology. Based on these data, primers were designed to clone the *amy*BL159 gene into expression vectors. The amylase gene, which includes the amino acid sequence of the native signal peptide, was cloned into expression vectors for heterologous production in *E. coli* and *B. subtilis* cells (Figures S2 and S3). The resulting recombinant plasmids carrying the *amy*BL159 gene were verified by sequencing (Evrogen, Moscow, Russia). Sequence analysis confirmed that the *amy*BL159 gene is 1539 base pairs long and encodes a polypeptide of 512 amino acid residues. The results of the multiple sequence alignment with known amylases are presented in Figure S2. In silico analysis of the amino acid sequence using the SignalP-6.0 program [36] predicted a signal peptide cleavage site between residues Ala26 and Ala27 (Figure S4).

### 3.3. Heterologous Expression and Purification of the Secreted Protein

**Figure 2:**
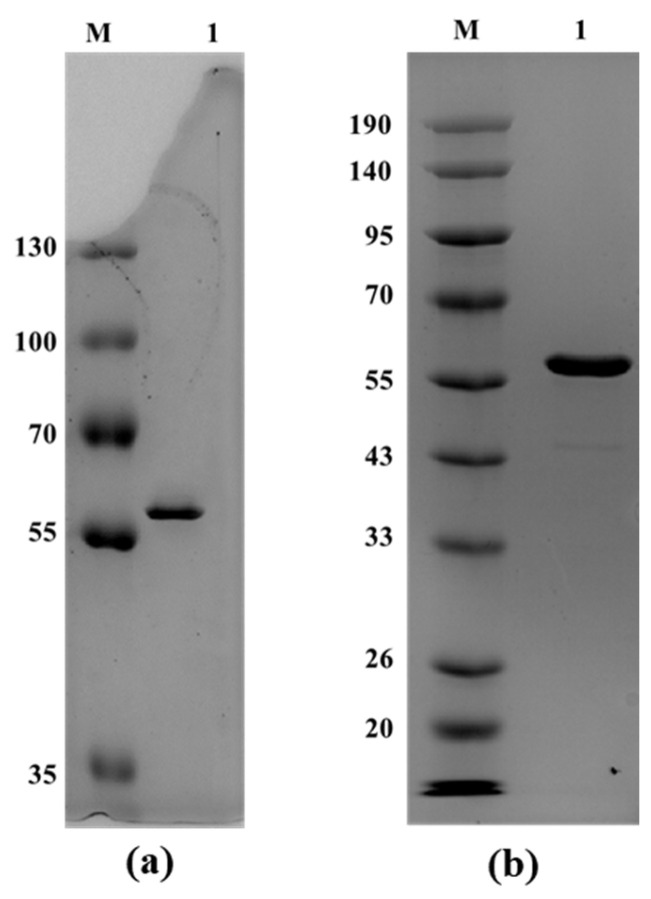
Electrophoretic analysis of purified recombinant amylases. (**a**) 12% SDS-polyacrylamide gel. M—protein marker (PageRuler™ Plus Prestained Protein Ladder, 10–250 kDa, ThermoFisher Scientific, Waltham, MA, USA); 1—purified AmyBL159_Ec_ preparation (expressed in *E. coli* cells); (**b**) 12% SDS-polyacrylamide gel. M—protein marker (Blue Plus V Protein Marker, 10–190 kDa, TransGen Biotech, Beijing, China); 1—purified AmyBL159_Bs_ preparation (expressed in *B. subtilis* cells). A total of 5 μg of purified protein preparation was loaded per lane. Molecular weights of marker proteins are indicated in kDa.

The amylase gene was successfully expressed in *E. coli* and *B. subtilis* cells using the pET22b and pHT01 expression vectors, respectively. The AmyBL159_Ec_ and AmyBL159_Bs_ proteins were purified from the culture supernatant of *E. coli* and *B. subtilis* by Ni-NTA metal-chelate chromatography facilitated by a fused 6xHis-tag at the C-terminus of the proteins. The protein concentration was determined spectrophotometrically at 280 nm. The purification yielded ≈2 mg of the α-amylase AmyBL159_Ec_ per liter of *E. coli* culture supernatant and approximately 3 mg of AmyBL159_Bs_ per liter of *B. subtilis* culture supernatant. The results of one step purification of the secreted protein are present on Figure 2.

The protein samples were aliquoted and stored at −80 °C for long-term preservation and further use in all subsequent experiments.

### 3.4. Temperature Optimum of Activity and Thermostability of Recombinant α-Amylases

#### 3.4.1. Determination of the Reaction Temperature Optimum

**Figure 3:**
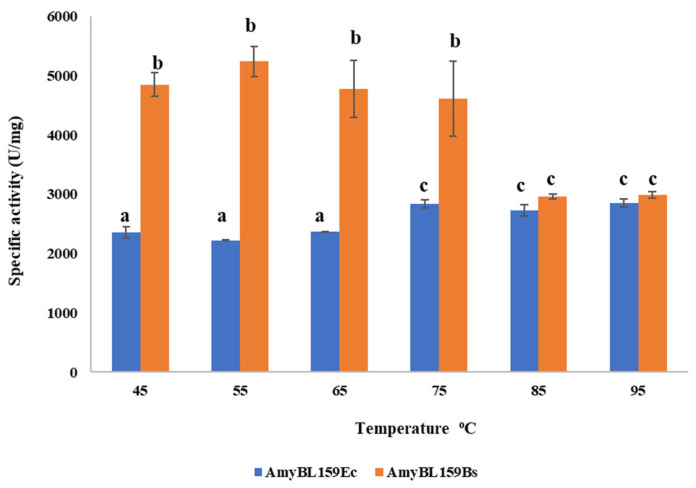
Activity profile of AmyBL159_Ec_, purified from *E. coli* culture supernatant, and AmyBL159_Bs_, produced in *B. subtilis* 168 cells, as a function of enzymatic reaction temperature. Data represent mean values ± standard deviation from three independent experiments. Bars indicated mean ± SD (n = three biological replicates). Columns marked with the different letters represent statistically significant difference.

The results of determination of the specific enzyme activity at different temperatures are presented in Figure 3. The recombinant α-amylase AmyBL159_Ec_ exhibited stable specific activity values over the range of 45–95 °C, with increasing of the activity at 75–95 °C (up to 2800 ± 74.1 U/mg). In contrast, the enzyme isolated from *B. subtillis* (AmyBL159_Bs)_ displayed maximum activity in the range of temperatures from 45 to 75 °C (up to 5200 ± 41 U/mg) but remained active within the 85–95 °C range at a level comparable to the maximum activity of AmyBL159_Ec_ (2984.6 ± 56.4 U/mg).

#### 3.4.2. Thermostability of the Recombinant α-Amylase

**Figure 4:**
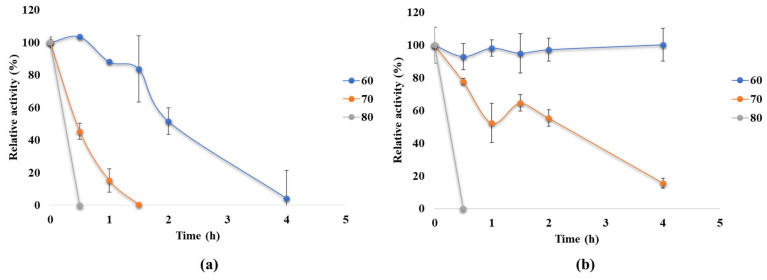
Thermostability profile of the recombinant amylase at different incubation temperatures: (**a**) AmyBL159_Ec_ and (**b**) AmyBL159_Bs_. The experiment was performed in triplicate under optimal reaction conditions. Bars indicated mean ± SD (n = three biological replicates).

The investigation of thermostability of the recombinant α-amylase variants from *B. licheniformis* MGMM159 (AmyBL159_Ec_ and AmyBL159_Bs_) revealed that the two enzymes exhibit distinct thermostability profiles, as illustrated in Figure 4.

As can be seen, the AmyBL159_Bs_ enzyme retained ≈100% of its activity during a 4-h incubation at 60 °C, whereas AmyBL159_Ec_ almost completely lost its activity activity over the same period, suggesting mis-folding or secretion artifacts. When the temperature was increased to 70 °C, both enzyme variants exhibited a decrease in activity within the first 2 h of incubation: AmyBL159_Ec_ lost full activity while AmyBL159_Bs_ lost ~50% (residual activity ~50%). This initial decline is likely associated with the loss of native conformation at the elevated temperature, particularly in the absence of stabilizing factors. The most pronounced loss of activity was observed at 80 °C, where both enzymes lost most of their activity within the first 30 min. The half-lives of AmyBL159_Ec_ and AmyBL159_Bs_ were: 2 h and more then 4 h at 60 °C, 30 min and 2.5 h at 70 °C, respectively, and about 15 min for both enzymes at 80 °C. A significant decrease in activity at higher temperatures was shown for some alpha-amylase enzymes from *Bacillus* species, which remain functional up to 70–75 °C but are rapidly deactivated at higher temperatures [37,38].

### 3.5. pH Optimum of Recombinant α-Amylase Forms from B. licheniformis MGMM159

**Figure 5:**
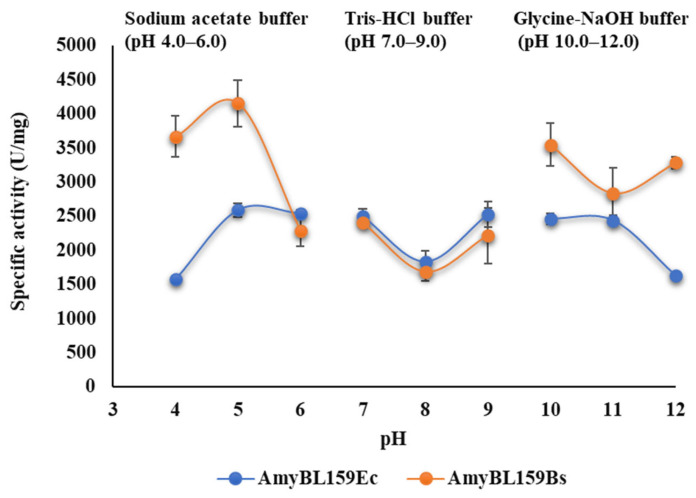
Influence of pH on the activity of purified AmyBL159_Ec_ and AmyBL159Bs amylase preparations. The experiment was performed in triplicate, and the mean values with standard deviations were determined. Bars indicated mean ± SD (n = three biological replicates).

The effect of pH on the activity of the recombinant α-amylase AmyBL159 was investigated across a range of pH 4.0–12.0 at a fixed temperature of 55 °C, which was established as the median optimal temperature for the catalytic activity of enzymes isolated from both expression hosts. Analysis of the data presented on Figure 5 revealed that both enzyme forms exhibited activity over a broad pH range, indicating their ability to function effectively under both acidic and alkaline conditions.

This property underscores the potential applicability of the recombinant *B. licheniformis* MGMM159 enzymes in industrial processes that require operation under extreme pH conditions.

### 3.6. Analysis of the Influence of Metal Cations, Chelating Agent, and Detergent on α-Amylase Activity

Studying the effects of metal cations, chelating agent and detergent on the activity of α-amylases is essential for understanding of the mechanisms that regulate their catalytic activity and stability. According to the literature, divalent cations such as Ca^2+^ and Mg^2+^ play a key role in maintaining the structural integrity of α-amylases, enhancing their thermostability and catalytic efficiency. Specifically, calcium ions stabilize the enzyme’s tertiary structure, preventing its denaturation at high temperatures, while Mg^2+^ helps maintain the enzyme’s optimal structural conformation [9,39,40,41]. In the present study, we examined the effects of various metal cations, EDTA and SDS on the activity of the recombinant α-amylases AmyBL159_Ec_ and AmyBL159_Bs_, expressed in *E. coli* and *B. subtilis*, respectively. The results, presented in Table 2, demonstrate that Mn^2+^ ions had the most pronounced stimulatory effect on enzyme activity, increasing it to 163% for AmyBL159_Ec_ and 142% for AmyBL159_Bs_. This may be attributed to the ability of Mn^2+^ to stabilize the enzyme structure by promoting its optimal conformation, which is consistent with previous studies [42]. Ca^2+^ and Mg^2+^ ions had a moderate effect on enzyme activity: Ca^2+^ increased the activity of AmyBL159Ec to 120%, while Mg^2+^ decreased it to 82%. For AmyBL159Bs, activity in the presence of Ca^2+^ was 96%, and in the presence of Mg^2+^, it was 75%. Possible reasons for this discrepancy could be unique structural features of the recombinant AmyBL159 α-amylase forms or specific experimental conditions, such as the influence of salt counterions.

It was demonstrated, that Na^+^ and K^+^ ions did not significantly affect the activity of AmyBL159_Bs_; however, Na^+^ ions reduced the activity of AmyBL159_Ec_ to 76%. The addition of the chelating agent EDTA led to a reduction in the enzyme activity, to 85% for AmyBL159_Ec_ and 68% for AmyBL159_Bs_. This underscores the role of divalent cations in maintaining the structural integrity and functional activity of the AmyBL159 α-amylase. The addition of the anionic detergent SDS caused a decrease in the enzyme activity to 50% (AmyBL159_Ec_) and 21% (AmyBL159_Bs_).

### 3.7. 3D-Model and Structural Properties of the α-Amylase AmyBL159

**Figure 6:**
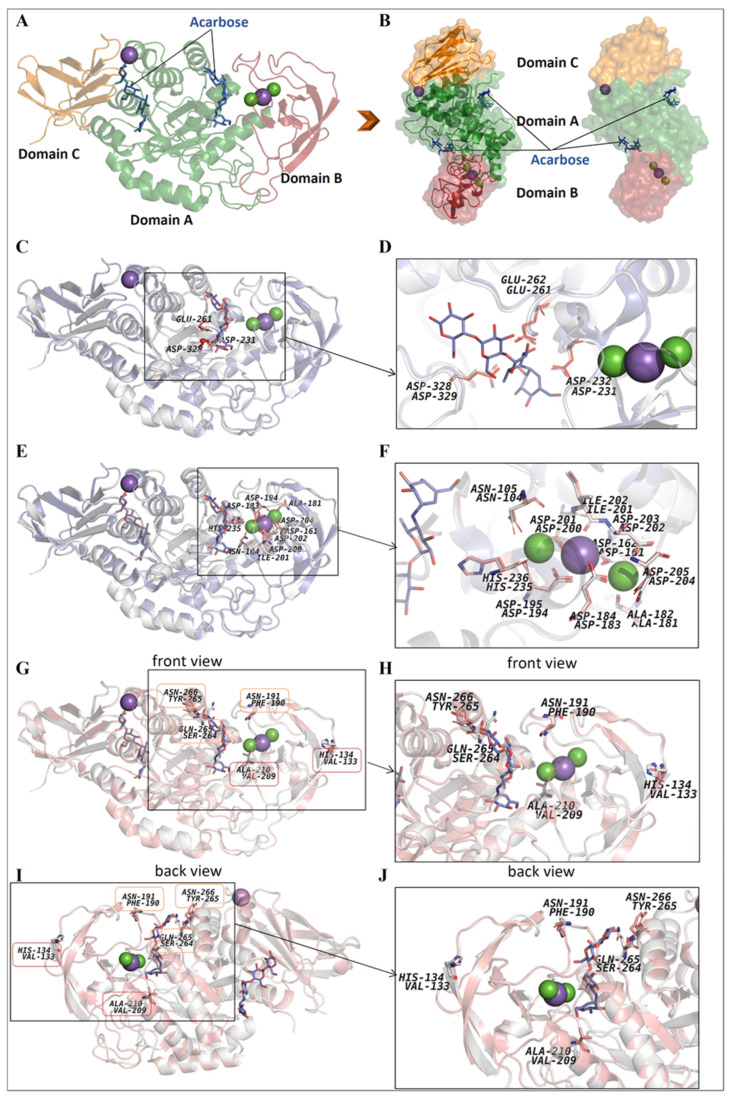
Structure properties of the α-amylase AmyBL159 (the model was predicted by AlphaFold 3): (**A**,**B**)—a domain organization in overall structure of the α-amylase AmyBL159; (**C**,**D**)—a superposition of the α-amylase AmyBL159 (carbon atoms are colored in gray) and α-amylase from *B. paralicheniformis* strain ATCC 9945a (RCSB PDB acc. no.: 6TOZ, carbon atoms are colored in blue) with presentation of the active cleft with catalytic residues (red color); (**E**,**F**)—a superposition of the α-amylase AmyBL159 (carbon atoms are colored in gray) and α-amylase from *B. paralicheniformis* strain ATCC 9945a (RCSB PDB acc. no.: 6TOZ, carbon atoms are colored in blue) with presentation of the Ca-Na-Ca triad; (**G**,**H**)—a superposition of the α-amylase AmyBL159 (carbon atoms are colored in gray) and α-amylase from *B. licheniformis* (RCSB PDB acc. no.: 1OB0, carbon atoms are colored in coral) with presentation of the mutation sites compared to wild type of the α-amylase (RCSB PDB acc. no.: 1BLI), the front view; (**I**,**J**)—the back view of the superposition of the α-amylase AmyBL159 (carbon atoms are colored in gray) and α-amylase from *B. licheniformis* (RCSB PDB acc. no.: 1OB0, carbon atoms are colored in coral) with presentation of the mutation sites compared to wild type of the α-amylase (RCSB PDB acc. no.: 1BLI). The molecule of substrate analogue (acarbose) presented in all structures is colored in blue. Other atoms as well as calcium (green sphere) and sodium (magenta sphere) ions are colored as element type.

The α-amylase AmyBL159 molecule, like most known α-amylases [43,44,45], consisted three domains (Figure 6A,B): (1) the catalytic domain A, mostly represented by a predominant α-helix structure, where the substrate is bound and catalytically cleaved, (2) the domain B, represented by a β-sheets, where two calcium atoms and one sodium atom are usually bound, tightly adjacent to the active cleft, and (3) the β-sheet domain C, where one sodium ion is most often located.

The crystal structure of the wild-type α-amylase from *B. licheniformis* (RCSB PDB acc. no.: 1BLI; [46] and the mutant form of the same enzyme (RCSB PDB acc. no.: 1OB0; [43]) was closest to the α-amylase AmyBL159 model in terms of amino acid composition (3 and 7 amino acid substitutions, correspondingly) (Figure 6, Table 3). Another enzyme with a known crystal structure similar to the α-amylases AmyBL159 was α-amylase from bacteria *B. paralicheniformis* strain ATCC 9945a (RCSB PDB acc. no.: 6TOZ; [45], where 20 amino acid substitutions were found. Like most α-amylases, the AmyBL159 molecule had a slightly asymmetrical shape, with cavities on both sides where, in other amylases, the substrate or analog molecules bind. Only one of the cavities contained strictly conserved residues involved in the cleavage of the carbohydrate chain (Figure 6C,D): two aspartate residues (Asp329 and Asp232 in AmyBL159 molecule corresponding to Asp328 and Asp321 in 6TOZ structure) and one glutamate residue (Glu262 in AmyBL159 molecule corresponding to Glu261 in 6TOZ structure).

**Figure 7:**
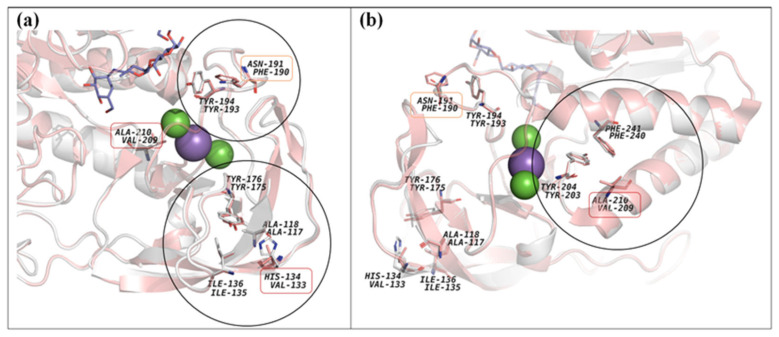
Potential hydrophobic interactions involving amino acid residues as a result of mutations at positions 133, 190, and 209 in α-amylase from *B. licheniformis* (RCSB PDB acc. no.: 1OB0, carbon atoms are colored in coral): (**a**)—front view, (**b**)—back view. Carbon atoms of the superposed α-amylase AmyBL159 structure are colored in gray. The molecule of substrate analogue (acarbose) is colored in blue. Other atoms as well as calcium (green sphere) and sodium (magenta sphere) ions are colored as element type.

Superposition of structures of the α-amylase AmyBL159 and the α-amylase from *B. paralicheniformis* strain ATCC 9945a revealed the presence of identical amino acids involved in the binding of the Ca-Na-Ca triad (Figure 6E,F) [45]: Asn105/Asn104, Asp162/Asp161, Ala182/Ala181, Asp184/Asp183, Asp195/Asp194, Asp201/200, Ile202/Ile201, Asp203/Asp202, Asp205/Asp204, His236/His235, correspondingly. This suggests the ability of the α-amylase AmyBL159 to bind a similar metal triad. Analysis of the effect of amino acid substitutions in a series of α-amylases of *B. licheniformis* on the thermal stability of the enzymes (Table 3, Figure 6I,J) showed an insignificant influence of mutation N190(191)F, Q264(265)S, N265(266)Y. At the same time, other mutations H133(134)V and A209(210)V seem to be more significant for increasing the thermal stability of the enzyme. A more detailed study of the amino acid environment at positions 133(134) and 209(210) in the α-amylase AmyBL159 and α-amylase from *B. paralicheniformis* strain ATCC 9945a suggested that substitutions of amino acids with hydrophobic ones will most likely strengthen the hydrophobic contacts in the region of the β-fold of domain B (in the case of H133(134)V) and between the α-helices of the domain A, which in turn stabilizes the overall structure of the enzyme and, consequently, leads to increased thermal stability (Figure 7). The amino acid substitution of asparagine at position 190 with hydrophobic phenylalanine does not contribute to the creation of more significant hydrophobic networks, since it is located in a random coil region.

Thus, we were able to clarify the significance of amino acid substitutions previously made using α-amylases from *B. licheniformis* [43,46], which opens up the prospect of creating biotechnologically valuable enzymes with desired properties.

## 4. Discussion

In this study, the heterologous expression of the α-amylase gene *amy*BL159 from the thermophilic strain *B. licheniformis* MGMM159 was successfully achieved in two different expression systems: *E. coli* BL21(DE3)pLys and *B. subtilis* 168. The selection of these systems is justified by their widespread use in biotechnology for recombinant protein production. *E. coli* is one of the most well-studied and versatile expression hosts, whereas *B. subtilis* has GRAS status, making it preferable for producing enzymes intended for food and industrial applications. Both recombinant enzymes (AmyBL159_Ec_ and AmyBL159_Bs_), were successfully secreted into the culture supernatant by both *E. coli* and *B. subtilis* using their native signal peptide, which significantly simplified the downstream purification process. In the literature described the expression of α-amylase from *B. licheniformis* and *B. laterosporus* in *E. coli* and its efficient secretion into the culture medium using its native signal peptide [47,48]. Using metal-chelate affinity chromatography on an Ni-NTA column resulted in a high degree of purification, yielding specific activities of 2224 ± 4.2 U/mg for AmyBL159_Ec_ and 5236 ± 249 U/mg for AmyBL159_Bs_. The higher specific activity of the enzyme expressed in *B. subtilis* may be attributed to correct protein folding and possible post-translational modifications, which occur more efficiently in Gram-positive bacteria [20,49]. Numerous studies in the current literature have investigated the properties of α-amylases isolated from various *B. licheniformis* strains and expressed in different heterologous systems. Table 4 summarizes the key properties of amylases isolated from various strains closely related bacterial.

Some of the described amylases exhibit a relatively narrow pH optimum and an elevated temperature optimum for functional activity. For instance, the amylase from strain *B. licheniformis* ATCC 27,811 (52 kDa), heterologously expressed in *E. coli* (with a specific activity of 584.61 U/mg), was shown to have an optimal pH of 8.0, while the enzyme lost 90% of its activity at pH 5.0 and 50% at pH 9.0. The temperature optimum for this amylase was around 70 °C [51]. In another study, an amylolytic enzyme from strain *B. licheniformis* LB04 (130 kDa) demonstrated maximum activity at pH 3.0 and a temperature of 80 °C, with a specific activity of the purified protein of 1851.7 U/mg [2]. The activity of the AmyBL159 amylase manifested differently depending on the expression host. The recombinant α-amylase AmyBL159_Ec_, expressed in *E. coli*, showed stable specific activity values within the range of 45–95 °C, varying from 2300 ± 4.2 U/mg to 2800 ± 74.1 U/mg. This is consistent with data reported in the literature for thermophilic α-amylases that retain activity at high temperatures [54]. In contrast, the AmyBL159_Bs_ enzyme isolated from *B. subtilis* was most active in the range of temperatures from 45–75 °C, which may be associated with specific features of protein processing in *B. subtilis*, as well as differences in protein folding and stabilization mechanisms between different host organisms. One of the most noteworthy observations in this study is the high functional activity of both recombinant AmyBL159 forms across a broad pH range. This property makes it potentially useful for application in biotechnological processes that occur under extreme pH conditions, such as detergent manufacturing or the food industry. Many researchers have reported on the influence of metal ions, detergents, and chelating substances on amylase activity. It is well-established that the activity of most amylases depends on metal ions, specifically divalent ions such as Ca^2+^, Mg^2+^, Mn^2+^, and Fe^2+^ [55]. For instance, Ca^2+^ has been reported to enhance the activity of α-amylase in the alkalophilic bacterium *Bacillus* sp. ANT-6 [24]. It is hypothesized that calcium ions play a role in regulating the structural stability and activity of α-amylase in *Bacillus* species. Calcium ions are likely to play a structural role, as they are located far from the active site and therefore cannot directly participate in catalysis. This is supported by research on the calcium-binding site in α-amylase from *Bacillus licheniformis* [56]. It was shown that α-amylases from *B. licheniformis* and *B. stearothermophilus* have two calcium-binding sites, Cal and CalI [57]. The CalI site is close to Cal, forming a triad with Na^+^ ions. This suggests that calcium ions may play a role in maintaining the stability of the enzyme’s structure. In the α-amylase of *B. licheniformis*, a triadic metal symmetry can be observed in the region surrounding Cal and CalI. Mutations that affect this region directly or indirectly typically have a significant impact on amylase activity at moderate temperatures [58]. Conversely, several studies have indicated that some amylases do not require calcium ions for their activity [37,59]. As is evident from our results (Table 2), the most pronounced stimulatory effect on enzyme activity was exerted by Mn^2+^, which increased activity to 163% for AmyBL159_Ec_ and 142% for AmyBL159_Bs_. This aligns with other studies demonstrating that Mn^2+^ can likely stabilize amylase structures [42,60,61]. In our work demonstrated that Ca^2+^ and Mg^2+^ ions did not have a significant stimulatory effect on the activity of AmyBL159_Bs_, which contradicts classical data on the role of these ions in stabilizing α-amylases. However, the addition of Ca^2+^ salts to the enzyme isolated from *E. coli* stimulated its activity by 20% (Table 2). The addition of EDTA led to a reduction in enzyme activity to 85% (*E. coli*) and 68% (*B. subtilis*), confirming the important role of divalent cations in maintaining enzyme function and indicating that their chelation is critical for these α-amylases. The addition of SDS to the reaction mixture caused a decrease in activity to 50% (*E. coli*) and 21% (*B. subtilis*), likely due to disruption of the protein’s tertiary structure. Data from our thermostability experiments demonstrated that AmyBL159_Bs_ is more stable at elevated temperatures at 60 °C and 70 °C but the both enzymes were more unstable under extreme conditions (80 °C). The differences in thermostability between the two enzyme variants may be linked to distinct folding pathways and post-translational modifications in the different expression systems, although similar data are not extensively reported in the literature.

The differences in the activity profiles of AmyBL159_Ec_ and AmyBL159_Bs_ under various pH levels and temperatures, as well as their contrasting thermostability, were surprising. Previous expression of an amylase from the *B. licheniformis* WX-02 strain in *E. coli* and *P. pastoris* did not report significant differences between the protein forms, despite a high level of glycosylation in the protein purified from *P. pastoris*. That study showed only minor differences in the thermostability of the glycosylated and non-glycosylated enzyme [54].

The performed calculation of the three-dimensional structure of the α-amylase AmyBL159 and subsequent structural-functional analysis carried out in a series of the known α-amylases from the bacteria of *B. licheniformis* made it possible to clarify the amino acid positions that affect the thermal stability of the α-amylases [43,46], which opens up the prospect of creating biotechnologically valuable enzymes with desired properties.

In summary, these findings expand our knowledge of amylases and emphasize the crucial role of the expression system in determining the functional characteristics of recombinant proteins. Moreover, this research provides a basis for the future improvement of AmyBL159 for industrial use.

## Figures and Tables

**Table 1:** Bacterial strains, vectors and recombinant plasmids.

Strain/Plasmid	Genotype/Features	Reference/Source
*E. coli* DH5α	F′ 80Δ*lac*Z M15 (*lac*ZYA-*arg*F) U169 *rec*A1 *end*A1*hsd*R17(rk−, mk+) *pho*A *sup*E44-*thi*-1 *gyr*A96 *rel*A1	[26]
*E. coli* BL21(DE3) pLys	F^−^*ompT hsdS*_B_(r_B_^−^ m_B_^−^)*gal dcm*(DE3) pLysS (Cm^R^)	Novagen
*B. subtilis* 168	wild type (GRAS)	[27,28]
pHT01	*E. coli*-*B. subtilis* shuttle vector, Amp^R^, Cm^R^, P_grac_-promoter	NovoProLab
pET22b	pBR322 *ori*, f1 *ori*, Amp^R^, P_T7_-promoter	Novagen
pET22b-amyBL159-6His	*amy*BL159 gene cloned in to *Nde*I и *Xho*I restriction sites of pET22b vector	This work
pHT01-amyBL159-6His	*amy*BL159 gene cloned in to *Bam*I и *Sma*I restriction sites of pHT01 vector	This work

**Table 2:** The effect of various compounds on the activity of AmyBL159_Ec_ and AmyBL159_Bs_ α-amylases. All values in the table represent means of three replicates with standard deviations under 5%.

Compound	Residual Specific Activity (%) of AmyBL159_Ec_	Residual Specific Activity (%) of AmyBL159_Bs_
Control *	100	100
NaCl (5 mM)	76	102
KCl (5 mM)	93	92
MnSO_4_ (5 mM)	163	142
MgSO_4_ (5 mM)	82	75
CaCl_2_ (5 mM)	120	96
SDS (1%)	50	21
EDTA (1 mM)	85	68

* Control—reaction mixture without adding of any agents.

**Table 3:** The effect of mutations on the temperature stability of recombinant α-amylase forms from *B. licheniformis*. The residues corresponding to the unchanged residues in the wild type of enzyme [43] are marked in green. Orange and red colors indicate mutations that are predicted to moderately and strongly increase enzyme stability, respectively.

	Enzyme Forms					
	Wild Type BLA *	Double Mutation *	Triple Mutation 1	Triple Mutation 2 (1BLI_A) *	Quadruple Mutation (1OB0_A) *	AmyBL159 (Position Corresponding to 1BLI_A or 1OB0_A)
Residues	H133	H133	V133	H133	V133	H134(133)
	L134	L134	L134	L134	L134	R135(134)
	N190	N190	F190	F190	F190	N190
	A209	A209	V209	A209	V209	A210(209)
	Q264	S264	Q264	S264	S264	Q265(264)
	N265	Y265	N265	Y265	Y265	N266(265)
	Q393	Q393	Q393	Q393	Q393	P394(393)
	A465	A465	A465	A465	E465	E466(465)
Stability, half-life, min	14 *	19 *	384 *	71 *	447 *	15 **

* at 85 °C [43]. ** at 80 °C (this work).

**Table 4:** Properties of recombinant α-amylases from *B. licheniformis* MGMM159 compared with other known amylases from closely related strains produced in various expression systems.

Strain-Donor of α-Amylase *B. licheniformis*	pH Optimum	T-Optimum, °C	Stability, Half-Live	GenBank Acc. No. (RCSB Acc. No.)	Host Strain for Expression	References
MGMM159	4.0–12.0	75–95	15 min at 80 °C	no info	*E. coli* BL21(DE3), secreted	This work
MGMM159	4.0–12.0	45–75	15 min at 80 °C	no info	*B. subtilis* 168, secreted	This work
no info *	no info	no info	71 min at 85 °C	1BLI_A (1BLI.pdb, muts N190F, Q264S and N265Y)	*E. coli* HB2151	[46,50]
no info	no info	no info	447 min at 85 °C	1OB0_A (1OB0.pdb, muts H133V; N190F; A209V; N264S, Q265Y)	*E.coli* HB2151	[43,50]
ATCC 27811	8.0	70	no info	no info	*E. coli* BL21-Codon Plus (DE3-RIPL)	[51]
ATCC 9945a	6.5	90	90 min at 80 °C	JN042159.1 (6TOY, 6TOZ, 6TP0, 6TP1, 6TP2)	*B. licheniformis* ATCC 9945a	[45,52]
NH1	5.0–10.0	90	40 min at 80 °C	ABL75259.1	*E. coli* BL21, secreted	[38]
RM44	5.0	100	>120 min at 80 °C	no info	*B. licheniformis* RM44	[53]
LB04	3.0	80	no info	no info	*B. licheniformis* LB04	[2]
WX-02	6.0–7.5	80	11 min at 70 °C	AKQ71831.1	*Pichia pastoris*	[54]
WX-02	6.0–7.6	80	15 min at 70 °C	AKQ71831.1	*E. coli* BL21 (DE3)	[54]

* no info—no information.

## Data Availability

The original contributions presented in this study are included in the article/Supplementary Materials. Further inquiries can be directed to the corresponding author.

## Supplementary Materials

The following supporting information can be downloaded at: https://www.mdpi.com/article/10.3390/microorganisms13122747/s1, Figure S1: Screening for amylase activity in isolates from wastewater sludge compost (Kazan, Tatarstan, Russia). The MGMM159 isolate is marked with an arrow.; Figure S2: Genetic map and annotated nucleotide sequence of the pET22-amyBL159 recombinant plasmid; Figure S3: Genetic map and annotated nucleotide sequence of the pHT01-amyBL159 recombinant plasmid; Figure S4: Multiple sequence alignment of amylase from closely related *B. licheniformis* strains. The AmyBL159 protein sequence utilized in this work is colored (orange). The signal peptide identified using SignalP 6.0 is indicated in bold type and gray shading.
