# Supplementary material for: Recombinant Forms of α-Amylase AmyBL159 from a Thermophilic Bacterium Bacillus licheniformis MGMM159: The Effect of the Expression System on the Enzyme Properties
Source: Microorganisms. 2025 Dec 2;13(12):2747. doi: 10.3390/microorganisms13122747 (PMC12735216; doi:10.3390/microorganisms13122747)
Supplement: Supplementary file 1 [file microorganisms-13-02747-s001.zip › microorganisms-4015270-supplementary.pdf]

Figure S1. Screening for amylase activity in isolates from wastewater sludge compost (Kazan, Tatarstan, Russia). The MGMM159 isolate is marked with an arrow.

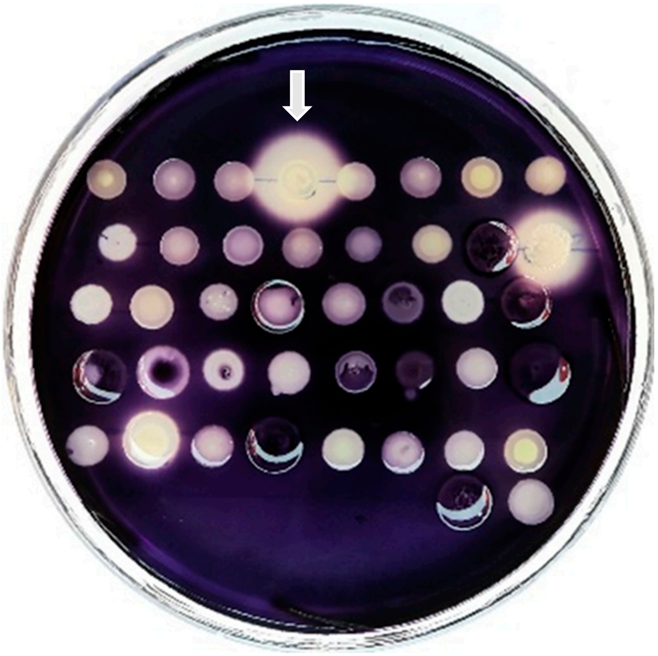

Figure S2. Genetic map and annotated nucleotide sequence of the pET22-amyBL159 recombinant plasmid.

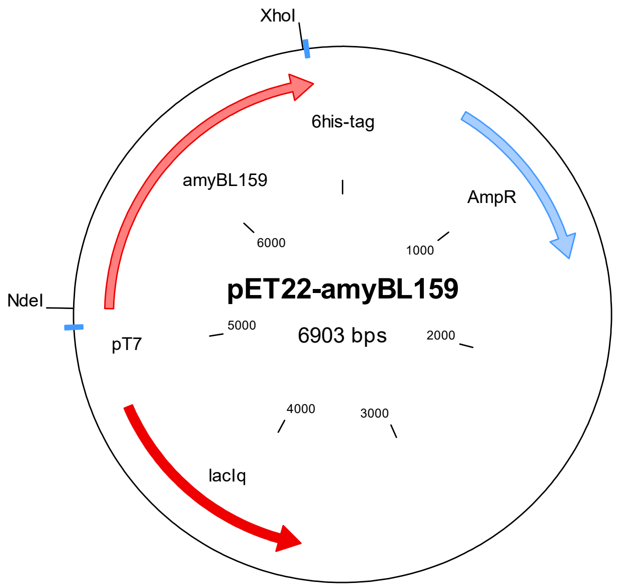


```
pET22-amyBL159          6903 bp    DNA     circular

REFERENCE    1  (bases 1 to 6903)
COMMENT      SECID/File created by Clone Manager, Scientific & Educational
Software
COMMENT      SECNOTES|Vector molecule:  i-pET22b*
                Fragment ends: XhoI and NdeI
                Fragment size: 5365
             Insert molecule:  amyBL-NdeI-XhoI
                Fragment ends: NdeI and XhoI
                Fragment size: 1538
FEATURES             Location/Qualifiers
     CDS             599..1459
                     /gene="AmpR"
                     /SECDrawAs="Gene"
                     /SECStyleId=1
     CDS             complement(3648..4730)
                     /gene="lacIq"
                     /SECDrawAs="Gene"
                     /SECStyleId=1
     misc_feature    5116..5133
                     /gene="pT7"
                     /SECDrawAs="Region"
                     /SECStyleId=1
     misc_feature    5136..5161
                     /gene="lac-operator"
                     /SECDrawAs="Label"
     misc_feature    5205..5291
                     /SECDrawAs="Label"
     CDS             5205..6766
                     /gene="amyBL159"
```

```
                     /SECDrawAs="Gene"
                     /SECStyleId=1
     misc_feature    6747..6764
                     /gene="6his-tag"
                     /SECDrawAs="Region"
                     /SECStyleId=1
ORIGIN
        1 tggcgaatgg gacgcgccct gtagcggcgc attaagcgcg gcgggtgtgg tggttacgcg
       61 cagcgtgacc gctacacttg ccagcgccct agcgcccgct cctttcgctt tcttcccttc
      121 ctttctcgcc acgttcgccg gctttccccg tcaagctcta aatcgggggc tccctttagg
      181 gttccgattt agtgctttac ggcacctcga ccccaaaaaa cttgattagg gtgatggttc
      241 acgtagtggg ccatcgccct gatagacggt ttttcgccct ttgacgttgg agtccacgtt
      301 ctttaatagt ggactcttgt tccaaactgg aacaacactc aaccctatct cggtctattc
      361 ttttgattta taagggattt tgccgatttc ggcctattgg ttaaaaaatg agctgattta
      421 acaaaaattt aacgcgaatt ttaacaaaat attaacgttt acaatttcag gtggcacttt
      481 tcggggaaat gtgcgcggaa cccctatttg tttatttttc taaatacatt caaatatgta
      541 tccgctcatg agacaataac cctgataaat gcttcaataa tattgaaaaa ggaagagtat
      601 gagtattcaa catttccgtg tcgcccttat tccctttttt gcggcatttt gccttcctgt
      661 ttttgctcac ccagaaacgc tggtgaaagt aaaagatgct gaagatcagt tgggtgcacg
      721 agtgggttac atcgaactgg atctcaacag cggtaagatc cttgagagtt ttcgccccga
      781 agaacgtttt ccaatgatga gcacttttaa agttctgcta tgtggcgcgg tattatcccg
      841 tattgacgcc gggcaagagc aactcggtcg ccgcatacac tattctcaga atgacttggt
      901 tgagtactca ccagtcacag aaaagcatct tacggatggc atgacagtaa gagaattatg
      961 cagtgctgcc ataaccatga gtgataacac tgcggccaac ttacttctga caacgatcgg
     1021 aggaccgaag gagctaaccg cttttttgca caacatgggg gatcatgtaa ctcgccttga
     1081 tcgttgggaa ccggagctga atgaagccat accaaacgac gagcgtgaca ccacgatgcc
     1141 tgcagcaatg gcaacaacgt tgcgcaaact attaactggc gaactactta ctctagcttc
     1201 ccggcaacaa ttaatagact ggatggaggc ggataaagtt gcaggaccac ttctgcgctc
     1261 ggcccttccg gctggctggt ttattgctga taaatctgga gccggtgagc gtgggtctcg
     1321 cggtatcatt gcagcactgg ggccagatgg taagccctcc cgtatcgtag ttatctacac
     1381 gacggggagt caggcaacta tggatgaacg aaatagacag atcgctgaga taggtgcctc
     1441 actgattaag cattggtaac tgtcagacca agtttactca tatatacttt agattgattt
     1501 aaaacttcat ttttaattta aaaggatcta ggtgaagatc ctttttgata atctcatgac
     1561 caaaatccct taacgtgagt tttcgttcca ctgagcgtca gaccccgtag aaaagatcaa
     1621 aggatcttct tgagatcctt tttttctgcg cgtaatctgc tgcttgcaaa caaaaaaacc
     1681 accgctacca gcggtggttt gtttgccgga tcaagagcta ccaactcttt ttccgaaggt
     1741 aactggcttc agcagagcgc agataccaaa tactgtcctt ctagtgtagc cgtagttagg
     1801 ccaccacttc aagaactctg tagcaccgcc tacatacctc gctctgctaa tcctgttacc
     1861 agtggctgct gccagtggcg ataagtcgtg tcttaccggg ttggactcaa gacgatagtt
     1921 accggataag gcgcagcggt cgggctgaac ggggggttcg tgcacacagc ccagcttgga
     1981 gcgaacgacc tacaccgaac tgagatacct acagcgtgag ctatgagaaa gcgccacgct
     2041 tcccgaaggg agaaaggcgg acaggtatcc ggtaagcggc agggtcggaa caggagagcg
     2101 cacgagggag cttccagggg gaaacgcctg gtatctttat agtcctgtcg ggtttcgcca
     2161 cctctgactt gagcgtcgat ttttgtgatg ctcgtcaggg gggcggagcc tatggaaaaa
     2221 cgccagcaac gcggcctttt tacggttcct ggccttttgc tggccttttg ctcacatgtt
     2281 ctttcctgcg ttatcccctg attctgtgga taaccgtatt accgcctttg agtgagctga
     2341 taccgctcgc cgcagccgaa cgaccgagcg cagcgagtca gtgagcgagg aagcggaaga
     2401 gcgcctgatg cggtattttc tccttacgca tctgtgcggt atttcacacc gcatatatgg
     2461 tgcactctca gtacaatctg ctctgatgcc gcatagttaa gccagtatac actccgctat
     2521 cgctacgtga ctgggtcatg gctgcgcccc gacacccgcc aacacccgct gacgcgccct
     2581 gacgggcttg tctgctcccg gcatccgctt acagacaagc tgtgaccgtc tccgggagct
     2641 gcatgtgtca gaggttttca ccgtcatcac cgaaacgcgc gaggcagctg cggtaaagct
     2701 catcagcgtg gtcgtgaagc gattcacaga tgtctgcctg ttcatccgcg tccagctcgt
     2761 tgagtttctc cagaagcgtt aatgtctggc ttctgataaa gcgggccatg ttaagggcgg
     2821 ttttttcctg tttggtcact gatgcctccg tgtaaggggg atttctgttc atgggggtaa
     2881 tgataccgat gaaacgagag aggatgctca cgatacgggt tactgatgat gaacatgccc
     2941 ggttactgga acgttgtgag ggtaaacaac tggcggtatg gatgcggcgg gaccagagaa
     3001 aaatcactca gggtcaatgc cagcgcttcg ttaatacaga tgtaggtgtt ccacagggta
     3061 gccagcagca tcctgcgatg cagatccgga acataatggt gcagggcgct gacttccgcg
     3121 tttccagact ttacgaaaca cggaaaccga agaccattca tgttgttgct caggtcgcag
     3181 acgttttgca gcagcagtcg cttcacgttc gctcgcgtat cggtgattca ttctgctaac
     3241 cagtaaggca accccgccag cctagccggg tcctcaacga caggagcacg atcatgcgca
     3301 cccgtggggc cgccatgccg gcgataatgg cctgcttctc gccgaaacgt ttggtggcgg
     3361 gaccagtgac gaaggcttga gcgagggcgt gcaagattcc gaataccgca agcgacaggc
     3421 cgatcatcgt cgcgctccag cgaaagcggt cctcgccgaa aatgacccag agcgctgccg
     3481 gcacctgtcc tacgagttgc atgataaaga agacagtcat aagtgcggcg acgatagtca
     3541 tgccccgcgc ccaccggaag gagctgactg ggttgaaggc tctcaagggc atcggtcgag
     3601 atcccggtgc ctaatgagtg agctaactta cattaattgc gttgcgctca ctgcccgctt
     3661 tccagtcggg aaacctgtcg tgccagctgc attaatgaat cggccaacgc gcggggagag
     3721 gcggtttgcg tattgggcgc cagggtggtt tttcttttca ccagtgagac gggcaacagc
```

```
     3781 tgattgccct tcaccgcctg gccctgagag agttgcagca agcggtccac gctggtttgc
     3841 cccagcaggc gaaaatcctg tttgatggtg gttaacggcg ggatataaca tgagctgtct
     3901 tcggtatcgt cgtatcccac taccgagata tccgcaccaa cgcgcagccc ggactcggta
     3961 atggcgcgca ttgcgcccag cgccatctga tcgttggcaa ccagcatcgc agtgggaacg
     4021 atgccctcat tcagcatttg catggtttgt tgaaaaccgg acatggcact ccagtcgcct
     4081 tcccgttccg ctatcggctg aatttgattg cgagtgagat atttatgcca gccagccaga
     4141 cgcagacgcg ccgagacaga acttaatggg cccgctaaca gcgcgatttg ctggtgaccc
     4201 aatgcgacca gatgctccac gcccagtcgc gtaccgtctt catgggagaa aataatactg
     4261 ttgatgggtg tctggtcaga gacatcaaga aataacgccg gaacattagt gcaggcagct
     4321 tccacagcaa tggcatcctg gtcatccagc ggatagttaa tgatcagccc actgacgcgt
     4381 tgcgcgagaa gattgtgcac cgccgcttta caggcttcga cgccgcttcg ttctaccatc
     4441 gacaccacca cgctggcacc cagttgatcg gcgcgagatt taatcgccgc gacaatttgc
     4501 gacggcgcgt gcagggccag actggaggtg gcaacgccaa tcagcaacga ctgtttgccc
     4561 gccagttgtt gtgccacgcg gttgggaatg taattcagct ccgccatcgc cgcttccact
     4621 ttttcccgcg ttttcgcaga aacgtggctg gcctggttca ccacgcggga aacggtctga
     4681 taagagacac cggcatactc tgcgacatcg tataacgtta ctggtttcac attcaccacc
     4741 ctgaattgac tctcttccgg gcgctatcat gccataccgc gaaaggtttt gcgccattcg
     4801 atggtgtccg ggatctcgac gctctccctt atgcgactcc tgcattagga agcagcccag
     4861 tagtaggttg aggccgttga gcaccgccgc cgcaaggaat ggtgcatgca aggagatggc
     4921 gcccaacagt cccccggcca cggggcctgc caccataccc acgccgaaac aagcgctcat
     4981 gagcccgaag tggcgagccc gatcttcccc atcggtgatg tcggcgatat aggcgccagc
     5041 aaccgcacct gtggcgccgg tgatgccggc cacgatgcgt ccggcgtaga ggatcgagat
     5101 ctcgatcccg cgaaattaat acgactcact ataggggaat tgtgagcgga taacaattcc
     5161 cctctagaaa taattttgtt taactttaag aaggagatat acatatgaaa caacaaaaac
     5221 ggctttacgc ccgattgctg acgctgttat ttgcgctcat cttcttgctg cctcattctg
     5281 cagcagcggc ggcaaatctt aatgggacgc tgatgcagta ttttgaatgg tacatgccca
     5341 atgacggcca acattggaag cgtttgcaaa acgactcggc atatttggct gaacacggta
     5401 ttactgccgt ctggattccc ccggcatata agggaacgag ccaagcggat gtgggctacg
     5461 gtgcttacga cctttatgat ttaggggagt ttcatcaaaa agggacggtt cggacaaagt
     5521 acggcacaaa aggagagctg caatctgcga tcaaaagtct tcattcccgc gacattaacg
     5581 tttacgggga tgtggtcatc aaccacaaag gcggcgctga tgcgaccgaa gatgtaaccg
     5641 cggttgaagt cgatcccgct gaccgcaacc gcgtaatttc aggagaacac cgaattaaag
     5701 cctggacaca ttttcatttt ccggggcgcg gcagcacata cagcgatttt aaatggcatt
     5761 ggtaccattt tgacggaacc gattgggacg agtcccgaaa gctgaaccgc atctataagt
     5821 ttcaaggaaa ggcttgggat tgggaagttt ccaatgaaaa cggcaactat gattatttga
     5881 tgtatgccga catcgattat gaccatcctg atgtcgcagc agaaattaag agatggggca
     5941 cttggtatgc caatgaactg caattggacg gtttccgtct tgatgctgtc aaacacatta
     6001 aatttctttt tttgcgggat tgggttaatc atgtcaggga aaaaacgggg aaggaaatgt
     6061 ttacggtagc tgaatattgg cagaatgact tgggcgcgct ggaaaactat ttgaacaaaa
     6121 caaattttaa tcattcagtg tttgacgtgc cgcttcatta tcagttccat gctgcatcga
     6181 cacagggagg cggctatgat atgaggaaat tgctgaacgg tacggtcgtt tccaagcatc
     6241 cgttgaaatc ggttacattt gtcgataacc atgatacaca gccgggggcaa tcgcttgagt
     6301 cgactgtcca aacatggttt aagccgcttg cttacgcttt tattctcaca agggaatctg
     6361 gataccctca ggttttctac ggggatatgt acgggacgaa aggagactcc cagcgcgaaa
     6421 ttcctgcctt gaaacacaaa attgaaccga tcttaaaagc gagaaaaccg tatgcgtacg
     6481 gagcacagca tgattatttc gaccaccatg acattgtcgg ctggacaagg gaaggcgaca
     6541 gctcggttgc aaattcaggt ttggcggcat taataacaga cggacccggt ggggcaaagc
     6601 gaatgtatgt cggccggcaa aacgccggtg agacatggca tgacattacc ggaaaccgtt
     6661 cggagccggt tgtcatcaat tcggaaggct ggggagagtt tcacgtaaac ggcgggtcgg
     6721 tttcaattta tgttcaaaga ctcgagcacc accaccacca ccactgagat ccggctgcta
     6781 acaaagcccg aaaggaagct gagttggctg ctgccaccgc tgagcaataa ctagcataac
     6841 ccccttggggc ctctaaacgg gtcttgaggg gttttttgct gaaaggagga actatatccg
     6901 gat
//
```

Figure S3. Genetic map and annotated nucleotide sequence of the pHT01-amyBL159 recombinant plasmid

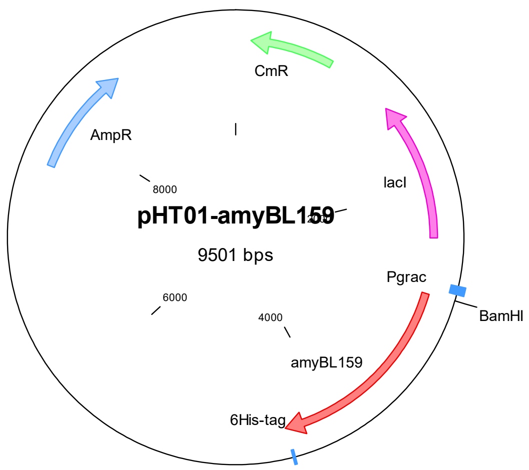

pHT01-amyBL159 9501 bp DNA

```
REFERENCE   1  (bases 1 to 9501)
COMMENT     SECID/File created by Clone Manager, Scientific & Educational
Software
COMMENT     SECNOTES|Vector molecule:  Fragment 2
               Fragment ends: XbaI and BamHI
               Fragment size: 7950
            Insert molecule:  amy-Bam-Xba
               Fragment ends: BamHI and XbaI
               Fragment size: 1545
FEATURES             Location/Qualifiers
     source          1..2804
                     /mol_type="other DNA"
                     /organism="synthetic DNA construct"
                     /SECDrawAs="Info only"
                     /SECName="source''"
     CDS             complement(116..763)
                     /gene="cat"
                     /label="Chloramphenicol acetyltransferase"
                     /note="Chloramphenicol acetyltransferase from
                     Staphylococcus aureus. Accession#: P00485"
                     /product="Chloramphenicol acetyltransferase from
                     Staphylococcus aureus. Accession#: P00485"
                     /SECDrawAs="Gene"
                     /SECStyleId=1
                     /SECName="CmR"
                     /SECDescr="Chloramphenicol acetyltransferase from
Staphylococcus aureus. Accession#: P00485"
     CDS             complement(1301..2380)
                     /label="lacI"
                     /note="lac repressor"
                     /SECDrawAs="Gene"
```

```
                     /SECStyleId=1
                     /SECName="lacI"
                     /SECDescr="lac repressor"
     misc_feature    2705..2761
                     /gene="Pgrac"
                     /SECDrawAs="Region"
                     /SECStyleId=1
     CDS             2810..4369
                     /gene="amyBL159"
                     /SECDrawAs="Gene"
                     /SECStyleId=1
     misc_feature    4350..4366
                     /gene="6His-tag"
                     /SECDrawAs="Region"
                     /SECStyleId=1
     source          4370..9501
                     /mol_type="other DNA"
                     /organism="synthetic DNA construct"
                     /SECDrawAs="Info only"
                     /SECName="'source"
     CDS             7683..8540
                     /label="AmpR"
                     /note="beta-lactamase"
                     /SECDrawAs="Gene"
                     /SECStyleId=1
                     /SECName="AmpR"
                     /SECDescr="beta-lactamase"
ORIGIN
        1 ttaagttatt ggtatgactg gttttaagcg caaaaaaagt tgctttttcg tacctattaa
       61 tgtatcgttt tagaaaaccg actgtaaaaa gtacagtcgg cattatctca tattataaaa
      121 gccagtcatt aggcctatct gacaattcct gaatagagtt cataaacaat cctgcatgat
      181 aaccatcaca aacagaatga tgtacctgta aagatagcgg taaatatatt gaattacctt
      241 tattaatgaa ttttcctgct gtaataatgg gtagaaggta attactatta ttattgatat
      301 ttaagttaaa cccagtaaat gaagtccatg gaataataga aagagaaaaa gcattttcag
      361 gtataggtgt tttgggaaac aatttccccg aaccattata tttctctaca tcagaaaggt
      421 ataaatcata aaactctttg aagtcattct ttacaggagt ccaaatacca gagaatgttt
      481 tagatacacc atcaaaaatt gtataaagtg gctctaactt atcccaataa cctaactctc
      541 cgtcgctatt gtaaccagtt ctaaaagctg tatttgagtt tatcaccctt gtcactaaga
      601 aaataaatgc agggtaaaat ttatatcctt cttgttttat gtttcggtat aaaacactaa
      661 tatcaatttc tgtggttata ctaaaagtcg tttgttggtt caaataatga ttaaatatct
      721 cttttctctt ccaattgtct aaatcaattt tattaaagtt catttgatat gcctcctaaa
      781 tttttatcta aagtgaattt aggaggctta cttgtctgct ttcttcatta gaatcaatcc
      841 tttttaaaa gtcaatatta ctgtaacata aatatatatt ttaaaaatat cccactttat
      901 ccaattttcg tttgttgaac taatgggtgc tttagttgaa gaataaaaga ccacattaaa
      961 aaatgtggtc ttttgtgttt ttttaaagga tttgagcgta gcgaaaaatc cttttctttc
     1021 ttatcttgat aataagggta actattgccg atcgtccatt ccgacagcat cgccagtcac
     1081 tatggcgtgc tgctagcgcc attcgccatt caggctgcgc aactgttggg aagggcgatc
     1141 ggtgcgggcc tcttcgctat tacgccagct ggcgaaaggg ggatgtgctg caaggcgatt
     1201 aagttgggta acgccagggt tttcccagtc acgacgttgt aaaacgacgg ccagtgaatt
     1261 cgagctcagg ccttaactca cattaattgc gttgcgctca ctgcccgctt tccagtcggg
     1321 aaacctgtcg tgccagctgc attaatgaat cggccaacgc gcggggagag gcggtttgcg
     1381 tattgggcgc cagggtggtt tttcttttca ccagtgagac gggcaacagc tgattgccct
     1441 tcaccgcctg gccctgagag agttgcagca agcggtccac gctggtttgc cccagcaggc
     1501 gaaaatcctg tttgatggtg gttaacggcg ggatataaca tgagctgtct tcggtatcgt
     1561 cgtatcccac taccgagata tccgcaccaa cgcgcagccc ggactcggta atggcgcgca
     1621 ttgcgcccag cgccatctga tcgttggcaa ccagcatcgc agtgggaacg atgccctcat
     1681 tcagcatttg catggtttgt tgaaaaccgg acatggcact ccagtcgcct tcccgttccg
     1741 ctatcggctg aatttgattg cgagtgagat atttatgcca gccagccaga cgcagacgcg
     1801 ccgagacaga acttaatggg cccgctaaca gcgcgatttg ctggtgaccc aatgcgacca
     1861 gatgctccac gcccagtcgc gtaccgtctt catgggagaa aataatactg ttgatgggtg
     1921 tctggtcaga gacatcaaga aataacgccg gaacattagt gcaggcagct tccacagcaa
     1981 tggcatcctg gtcatccagc ggatagttaa tgatcagccc actgacgcgt tgcgcgagaa
     2041 gattgtgcac cgccgcttta caggcttcga cgccgcttcg ttctaccatc gacaccacca
     2101 cgctggcacc cagttgatcg gcgcgagatt taatcgccgc gacaatttgc gacggcgcgt
     2161 gcagggccag actggaggtg gcaacgccaa tcagcaacga ctgtttgccc gccagttgtt
     2221 gtgccacgcg gttgggaatg taattcagct ccgccatcgc cgcttccact tttcccgcg
```

```
2281 ttttcgcaga aacgtggctg gcctggttca ccacgcggga aacggtctga taagagacac
2341 cggcatactc tgcgacatcg tataacgtta ctggtttcat caaaatcgtc tccctccgtt
2401 tgaatatttg attgatcgta accagatgaa gcactctttc cactatccct acagtgttat
2461 ggcttgaaca atcacgaaac aataattggt acgtacgatc tttcagccga ctcaaacatc
2521 aaatcttaca aatgtagtct ttgaaagtat tacatatgta agatttaaat gcaaccgttt
2581 tttcggaagg aaatgatgac ctcgtttcca ccggaattag cttggtacca gctattgtaa
2641 cataatcggt acgggggtga aaaagctaac ggaaaaggga gcggaaaaga atgatgtaag
2701 cgtgaaaaat tttttatctt atcacttgaa attggaaggg agattcttta ttataagaat
2761 tgtggaattg tgagcggata acaattccca attaaaggag gaaggatcca tgaaacaaca
2821 aaaacggctt tacgcccgat tgctgacgct gttatttgcg ctcatcttct tgctgcctca
2881 ttctgcagca gcggcggcaa atcttaatgg gacgctgatg cagtattttg aatggtacat
2941 gcccaatgac ggccaacatt ggaagcgttt gcaaaacgac tcggcatatt tggctgaaca
3001 cggtattact gccgtctgga ttcccccggc atataaggga acgagccaag cggatgtggg
3061 ctacggtgct tacgaccttt atgatttagg ggagtttcat caaaaaggga cggttcggac
3121 aaagtacggc acaaaaggag agctgcaatc tgcgatcaaa agtcttcatt cccgcgacat
3181 taacgtttac ggggatgtgg tcatcaacca caaaggcggc gctgatgcga ccgaagatgt
3241 aaccgcggtt gaagtcgatc ccgctgaccg caaccgcgta atttcaggag aacaccgaat
3301 taaagcctgg acacattttc attttccggg gcgcggcagc acatacagcg attttaaatg
3361 gcattggtac cattttgacg gaaccgattg ggacgagtcc cgaaagctga accgcatcta
3421 taagtttcaa ggaaaggctt gggattggga agtttccaat gaaaacggca actatgatta
3481 tttgatgtat gccgacatcg attatgacca tcctgatgtc gcagcagaaa ttaagagatg
3541 gggcacttgg tatgccaatg aactgcaatt ggacggtttc cgtcttgatg ctgtcaaaca
3601 cattaaattt tctttttgc gggattgggt taatcatgtc agggaaaaaa cggggaagga
3661 aatgtttacg gtagctgaat attggcagaa tgacttgggc gcgctggaaa actatttgaa
3721 caaaacaaat tttaatcatt cagtgtttga cgtgccgctt cattatcagt tccatgctgc
3781 atcgacacag ggaggcggct atgatatgag gaaattgctg aacggtacgg tcgtttccaa
3841 gcatccgttg aaatcggtta catttgtcga taaccatgat acacagccgg ggcaatcgct
3901 tgagtcgact gtccaaacat ggtttaagcc gcttgcttac gcttttattc tcacaaggga
3961 atctggatac cctcaggttt tctacgggga tatgtacggg acgaaaggag actcccagcg
4021 cgaaattcct gccttgaaac acaaaattga accgatctta aaagcgagaa aaccgtatgc
4081 gtacggagca cagcatgatt atttcgacca ccatgacatt gtcggctgga caagggaagg
4141 cgacagctcg gttgcaaatt caggtttggc ggcattaata acagacggac ccggtggggc
4201 aaagcgaatg tatgtcggcc ggcaaaacgc cggtgagaca tggcatgaca ttaccggaaa
4261 ccgttcggag ccggttgtca tcaattcgga aggctgggga gagtttcacg taaacggcgg
4321 gtcggtttca atttatgttc aaagagagca ccaccaccac caccactgac ccggggcagc
4381 ccgcctaatg agcgggcttt tttcacgtca cgcgtccatg gagatctttg tctgcaactg
4441 aaaagtttat accttacctg gaacaaatgg ttgaaacata cgaggctaat atcggcttat
4501 taggaatagt ccctgtacta ataaaatcag gtggatcagt tgatcagtat attttggacg
4561 aagctcggaa agaatttgga gatgacttgc ttaattccac aattaaatta agggaaagaa
4621 taaagcgatt tgatgttcaa ggaatcacgg aagaagatac tcatgataaa gaagctctaa
4681 aactattcaa taaccttaca atggaattga tcgaaagggt ggaaggttaa tggtacgaaa
4741 attaggggat ctacctagaa agccacaagg cgataggtca agcttaaaga acccttacat
4801 ggatcttaca gattctgaaa gtaaagaaac aacagaggtt aaacaaacag aaccaaaaag
4861 aaaaaaagca ttgttgaaaa caatgaaagt tgatgtttca atccataata agattaaatc
4921 gctgcacgaa attctggcag catccgaagg gaattcatat tacttagagg atactattga
4981 gagagctatt gataagatgg ttgagacatt acctgagagc caaaaaactt tttatgaata
5041 tgaattaaaa aaaagaacca acaaaggctg agacagactc caaacgagtc tgttttttta
5101 aaaaaaatat taggagcatt gaatatatat tagagaatta agaaagacat gggaataaaa
5161 atattttaaa tccagtaaaa atatgataag attatttcag aatatgaaga actctgtttg
5221 tttttgatga aaaaacaaac aaaaaaaatc cacctaacgg aatctcaatt taactaacag
5281 cggccaaact gagaagttaa atttgagaag ggaaaaggc ggatttatac ttgtatttaa
5341 ctatctccat tttaacattt tattaaaccc catacaagtg aaaatcctct tttacactgt
5401 tcctttaggt gatcgcggag ggacattatg agtgaagtaa acctaaaagg aaatacagat
5461 gaattagtgt attatcgaca gcaaaccact ggaaataaaa tcgccaggaa gagaatcaaa
5521 aaagggaaag aagaagttta ttatgttgct gaaacggaag agaagatatg gacagaagag
5581 caaataaaaa acttttcttt agacaaattt ggtacgcata taccttacat agaaggtcat
5641 tatacaatct taaataatta cttctttgat ttttggggct atttttttagg tgctgaagga
5701 attgcgctct atgctcacct aactcgttat gcatacggca gcaaagactt ttgctttcct
5761 agtctacaaa caatcgctaa aaaaatggac aagactcctg ttacagttag aggctacttg
5821 aaactgcttg aaaggtacgg ttttatttgg aaggtaaacg tccgtaataa aaccaaggat
5881 aacacagagg aatccccgat ttttaagatt agacgtaagg ttcctttgct ttcagaagaa
5941 cttttaaatg gaaaccctaa tattgaaatt ccagatgacg aggaagcaca tgtaaagaag
6001 gctttaaaaa aggaaaaaga gggtcttcca aaggttttga aaaaagagca cgatgaattt
6061 gttaaaaaaa tgatggatga gtcagaaaca attaatattc cagaggcctt acaatatgac
6121 acaatgtatg aagatatact cagtaaagga gaaattcgaa aagaaatcaa aaaacaaata
6181 cctaatccta caacatcttt tgagagtata tcaatgacaa ctgaagagga aaaagtcgac
6241 agtactttaa aaagcgaaat gcaaaatcgt gtctctaagc cttcttttga tacctggttt
6301 aaaaacacta agatcaaaat tgaaaataaa aattgtttat tacttgtacc gagtgaattt
6361 gcatttgaat ggattaagaa aagatattta gaaacaatta aaacagtcct tgaagaagct
6421 ggatatgttt tcgaaaaaat cgaactaaga aaagtgcaat aaactgctga agtatttcag
6481 cagttttttt tatttagaaa tagtgaaaaa aatataatca gggaggtatc aatatttaat
```

```
6541 gagtactgat ttaaatttat ttagactgga attaataatt aacacgtaga ctaattaaaa
6601 tttaatgagg gataaagagg atacaaaaat attaatttca atccctatta aattttaaca
6661 aggggggat taaaatttaa ttagaggttt atccacaaga aaagacccta ataaaatttt
6721 tactagggtt ataacactga ttaatttctt aatgggggag ggattaaaat ttaatgacaa
6781 agaaaacaat cttttaagaa aagcttttaa aagataataa taaaaagagc tttgcgatta
6841 agcaaaactc tttacttttt cattgacatt atcaaattca tcgatttcaa attgttgttg
6901 tatcataaag ttaattctgt tttgcacaac cttttcagga atataaaaca catctgaggc
6961 ttgttttata aactcagggt cgctaaagtc aatgtaacgt agcatatgat atggtatagc
7021 ttccacccaa gttagccttt ctgcttcttc tgaatgtttt tcatatactt ccatgggtat
7081 ctctaaatga ttttcctcat gtagcaaggt atgagcaaaa agtttatgga attgatagtt
7141 cctctctttt tcttcaactt ttttatctaa aacaaacact ttaacatctg agtcaatgta
7201 agcataagat gtttttccag tcataatttc aatcccaaat cttttagaca gaaattctgg
7261 acgtaaatct tttggtgaaa gaattttttt atgtagcaat atatccgata cagcaccttc
7321 taaaagcgtt ggtgaatagg gcattttacc tatctcctct cattttgtgg aataaaaata
7381 gtcatattcg tccatctacc tatcctatta tcgaacagtt gaacttttta atcaaggatc
7441 agtccttttt ttcattattc ttaaactgtg ctcttaactt taacaactcg atttgttttt
7501 ccagatctcg agggtaacta gcctcgccga tcccgcaaga ggcccggcag tcaggtggca
7561 cttttcgggg aaatgtgcgc ggaaccccta tttgtttatt tttctaaata cattcaaata
7621 tgtatccgct catgagacaa taaccctgat aaatgcttca ataatattga aaaaggaaga
7681 gtatgagtat tcaacatttc cgtgtcgccc ttattccctt ttttgcggca ttttgccttc
7741 ctgtttttgc tcacccagaa acgctggtga aagtaaaaga tgctgaagat cagttgggtg
7801 cacgagtggg ttacatcgaa ctggatctca acagcggtaa gatccttgag agttttcgcc
7861 ccgaagaacg ttttccaatg atgagcactt ttaaagttct gctatgtggc gcggtattat
7921 cccgtattga cgccgggcaa gagcaactcg gtcgccgcat acactattct cagaatgact
7981 tggttgagta ctcaccagtc acagaaaagc atcttacgga tggcatgaca gtaagagaat
8041 tatgcagtgc tgccataacc atgagtgata acactgcggc caacttactt ctgacaacga
8101 tcggaggacc gaaggagcta accgcttttt tgcacaacat gggggatcat gtaactcgcc
8161 ttgatcgttg ggaaccggag ctgaatgaag ccataccaaa cgacgagcgt gacaccacga
8221 tgcctgtagc aatggcaaca acgttgcgca aactattaac tggcgaacta cttactctag
8281 cttcccggca acaattaata gactggatgg aggcggataa agttgcagga ccacttctgc
8341 gctcggccct tccggctggc tggtttattg ctgataaatc tggagccggt gagcgtgggt
8401 ctcgcggtat cattgcagca ctggggccag atggtaagcc ctcccgtatc gtagttatct
8461 acacgacggg gagtcaggca actatggatg aacgaaatag acagatcgct gagataggtg
8521 cctcactgat taagcattgg taactgtcag accaagttta ctcatatata ctttagattg
8581 atttaaaact tcatttttaa tttaaaagga tctaggtgaa gatccttttt gataatctca
8641 tgaccaaaat cccttaacgt gagttttcgt tccactgagc gtcagacccc gtagaaaaga
8701 tcaaaggatc ttcttgagat cctttttttc tgcgcgtaat ctgctgcttg caaacaaaaa
8761 aaccaccgct accagcggtg gtttgtttgc cggatcaaga gctaccaact ctttttccga
8821 aggtaactgg cttcagcaga gcgcagatac caaatactgt ccttctagtg tagccgtagt
8881 taggccacca cttcaagaac tctgtagcac cgcctacata cctcgctctg ctaatcctgt
8941 taccagtggc tgctgccagt ggcgataagt cgtgtcttac cgggttggac tcaagacgat
9001 agttaccgga taaggcgcag cggtcgggct gaacgggggg ttcgtgcaca cagcccagct
9061 tggagcgaac gacctacacc gaactgagat acctacagcg tgagctatga gaaagcgcca
9121 cgcttcccga agggagaaag gcggacaggt atccggtaag cggcagggtc ggaacaggag
9181 agcgcacgag ggagcttcca gggggaaacg cctggtatct ttatagtcct gtcgggtttc
9241 gccacctctg acttgagcgt cgatttttgt gatgctcgtc aggggggcgg agcctatgga
9301 aaaacgccag caacgcggcc tttttacggt tcctggcctt ttgctggcct tttgctcaca
9361 tgttctttcc tgcgttatcc cctgattctg tggataaccg tattaccgcc tttgagtgag
9421 ctgataccgc tcgccgcagc cgaacgaccg agcgcagcga gtcagtgagc gaggaagcgg
9481 aagagcgccc aatacgcatg c
//
```

Figure S4. Multiple sequence alignment of amylase from closely related *B. licheniformis* strains. The AmyBL159 protein sequence utilized in this work is colored (orange). The signal peptide identified using SignalP 6.0 is indicated in bold type and gray shading

```
WP_268400763.1      MKQQKRLYARLLPLLFALIFLLPHSAAAAANLKGTLMQYFEWYMPNDGQHWKRLQNDSAY

AMJ27400.1          ----------------------PHSAAAAANLNGTLMQYFEWYMPNDGQHWKRLQNDSAY

amyBL159            MKQQKRLYARLLTLLFALIFLLPHSAAAAANLNGTLMQYFEWYMPNDGQHWKRLQNDSAY

ABW90124.1          ---------------------------AAANLNGTLMQYFEWYMPNDGQHWKRLQNDSAY

ACN88151.1          MKQQKRLYARLLTLLFALIFLLPHSAAAAANLNGTLMQYFEWYMPNDGQHWKRLQNDSAY

1BLI_A              -----------------------------ANLNGTLMQYFEWYMPNDGQHWKRLQNDSAY
```

```
1OB0_A            -----------------------------ANLNGTLMQYFEWYMPNDGQHWKRLQNDSAY
ABW94981.1        ------------------------------------MQYFEWYMPNDGQHWKRLQNDSAY
1VJS_A            -----------------------------ANLNGTLMQYFEWYMPNDGQHWKRLQNDSAY
6TOY_A            -----------------------------ASLNGTLMQYFEWYMPNDGQHWKRLQNDSAY
                                                    ************************

WP_268400763.1    LAEHGITAVWIPPAYKGTSQADVGYGAYDLYDLGEFHQKGTVRTKYGTKGELQSAIKSLH
AMJ27400.1        LAEHGITAVWIPPAYKGTSQADVGYGAYDLYDLGEFHQKGTVRTKYGTKGELQSAIKSLH
amyBL159          LAEHGITAVWIPPAYKGTSQADVGYGAYDLYDLGEFHQKGTVRTKYGTKGELQSAIKSLH
ABW90124.1        LAEHGITAVWIPPAYKGTSQADVGYGAYDLYDLGEFHQKGTVRTKYSTKGELQSAIKSLH
ACN88151.1        LAEHGITAVWIPPAYKGTSQADVGYGAYDLYDLGEFHQKGTVRTKYSTKGELQSAIKSLH
1BLI_A            LAEHGITAVWIPPAYKGTSQADVGYGAYDLYDLGEFHQKGTVRTKYGTKGELQSAIKSLH
1OB0_A            LAEHGITAVWIPPAYKGTSQADVGYGAYDLYDLGEFHQKGTVRTKYGTKGELQSAIKSLH
ABW94981.1        LAEHGITAVWIPPAYKGTSQADVGYGAYDLYDLGEFHQKGTVRTKYGTKGELQSAIKSLH
1VJS_A            LAEHGITAVWIPPAYKGTSQADVGYGAYDLYDLGEFHQKGTVRTKYGTKGELQSAIKSLH
6TOY_A            LAEHGITAVWIPPAYKGTSQDDVGYGAYDLYDLGEFHQKGTVRTKYGTKGELQSAINSLH
                  ******************** *************************.*********:***

WP_268400763.1    SRDINVYGDVVINHKGGADATEDVTAVEVDPADRNRVISGEHRIKAWTHFHFPGRGSTYS
AMJ27400.1        SRDINVYGDVVINHKGGADATEDVTAVEVDPADRNRVISGEHRIKAWTHFHFPGRGSTYS
amyBL159          SRDINVYGDVVINHKGGADATEDVTAVEVDPADRNRVISGEHRIKAWTHFHFPGRGSTYS
ABW90124.1        SRDINVYGDVVINHKGGADATEDVTAVEVDPADRNRVISGEHRIKAWTHFHFPGRGSTYS
ACN88151.1        SRDINVYGDVVINHKGGADATEDVTAVEVDPADRNRVISGEHRIKAWTHFHFPGRGSTYS
1BLI_A            SRDINVYGDVVINHKGGADATEDVTAVEVDPADRNRVISGEHLIKAWTHFHFPGRGSTYS
1OB0_A            SRDINVYGDVVINHKGGADATEDVTAVEVDPADRNRVISGEVLIKAWTHFHFPGRGSTYS
ABW94981.1        SRDINVYGDVVITTKGGADATEDVTAVVVDPADRNRVISGEHLIKAWTHFHFPGRGSTYS
1VJS_A            SRDINVYGDVVINHKGGADATEDVTAVEVDPADRNRVISGEHLIKAWTHFHFPGRGSTYS
6TOY_A            SRDINVYGDVVINHKGGADATEYVTAVEVDPADRNRVTSGEQRIKAWTHFQFPGRGSTYS
                  ************. ******** **** ********* ***  *******:*********

WP_268400763.1    DFKWHWYHFDGTDWDESRKLNRIYKFQGKAWDWEVSNENGNYDYLMYADIDYDHPDVAAE
AMJ27400.1        DFKWHWYHFDGTDWDESRKLNRIYKFQGKAWDWEVSNENGNYDYLMYADIDYDHPDVAAE
amyBL159          DFKWHWYHFDGTDWDESRKLNRIYKFQGKAWDWEVSNENGNYDYLMYADIDYDHPDVAAE
ABW90124.1        DFKWHWYHFDGTDWDESRKLNRIYKFQGKAWDWEVSNENGNYDYLMYADIDYDHPDVAAE
ACN88151.1        DFKWHWYHFDGTDWDESRKLNRIYKFQGKAWDWEVSNENGNYDYLMYADIDYDHPDVAAE
1BLI_A            DFKWHWYHFDGTDWDESRKLNRIYKFQGKAWDWEVSNEFGNYDYLMYADIDYDHPDVAAE
1OB0_A            DFKWHWYHFDGTDWDESRKLNRIYKFQGKAWDWEVSNEFGNYDYLMYADIDYDHPDVVAE
ABW94981.1        DFKWHWYHFDGTDWDESRKLNRIYKFQGKAWDWEVSNENGNYDYLMYADIDYDHPDVAAE
```

1VJS_A	DFKWHWYHFDGTDWDESRKLNRIYKFQGKAWDWEVSNENGNYDYLMYADIDYDHPDVAAE
6TOY_A	DFKWYWYHFDGTDWDESRKLNRIYKFQGKAWDWEVSNENGNYDYLMYADIDYDHPDVTAE
	****:********************************* ******************.**

WP_268400763.1	IKRWGTWYANELQLDGFRLDAVKHIKFSFLRDWVNHVREKTGKEMFTVAEYWQNDLGALE
AMJ27400.1	IKRWGTWYANELQLDGFRLDAVKHIKFSFLRDWVNHVREKTGKEMFTVAEYWQNDLGALE
**amyBL159**	**IKRWGTWYANELQLDGFRLDAVKHIKFSFLRDWVNHVREKTGKEMFTVAEYWQNDLGALE**
ABW90124.1	IKRWGTWYANELQLDGFRLDAVKHIKFSFLRDWVNHVREKTGKEMFTVAEYWQNDLGALE
ACN88151.1	IKRWGTWYANELQLDGFRLDAVKHIKFSFLRDWVNHVREKTGKEMFTVAEYWQNDLGALE
1BLI_A	IKRWGTWYANELQLDGFRLDAVKHIKFSFLRDWVNHVREKTGKEMFTVAEYWSYDLGALE
1OB0_A	IKRWGTWYANELQLDGFRLDAVKHIKFSFLRDWVNHVREKTGKEMFTVAEYWSYDLGALE
ABW94981.1	IKRWGTWYANELLLDGFRLDAVKHIKFSFLRDWVNHVREKTGKEMFTVAEYWQNDLGALE
1VJS_A	IKRWGTWYANELQLDGFRLDAVKHIKFSFLRDWVNHVREKTGKEMFTVAEYWQNDLGALE
6TOY_A	IKRWGTWYANELQLDGFRLDAVKHIKFSFLRDWVNHVREKTGKEMFTVAEYWQNDLGALE
	************ ***************************************. ******

WP_268400763.1	NYLNKTNFNHSVFDVPLHYQFHAASTQGGGYDMRKLLNGTVVSKHPLKAVIFVDNHDTQP
AMJ27400.1	NYLNKTNFNHSVFDVPLHYQFHAASTQGGGYDMRKLLNSTVVSKHPLKAVTFVDNHDTQP
**amyBL159**	**NYLNKTNFNHSVFDVPLHYQFHAASTQGGGYDMRKLLNGTVVSKHPLKSVTFVDNHDTQP**
ABW90124.1	NYLNKTNFNHSVFDVPLHYQFHAASTQGGGYDMRKLLNGTVVSKHPLKAVTFVDNHDTQP
ACN88151.1	NYLNKTNFNHSVFDVPLHYQFHAASTQGGGYDMRKLLNGTVVSKHPLKAVTFVDNHDTQP
1BLI_A	NYLNKTNFNHSVFDVPLHYQFHAASTQGGGYDMRKLLNGTVVSKHPLKSVTFVDNHDTQP
1OB0_A	NYLNKTNFNHSVFDVPLHYQFHAASTQGGGYDMRKLLNGTVVSKHPLKSVTFVDNHDTQP
ABW94981.1	NYLNKTNFNHSVFDVPLHYQFHAASTQEGGYDMRKLLIGTVVSKHPLKSVTFVDNHDTQP
1VJS_A	NYLNKTNFNHSVFDVPLHYQFHAASTQGGGYDMRKLLNSTVVSKHPLKAVTFVDNHDTQP
6TOY_A	NYLNKTNFNHSVFDVPLHYQFHAASTQGGGYDMRKLLNGTVVSKHPVKAVTFVDNHDTQP
	*************************** ********* .*******:*:* *********

WP_268400763.1	GQSLESTVQTWFKPLAYAFILTRESGYPQVFYGDMYGTKGDSQREIPALKHKIEPILKAR
AMJ27400.1	GQSLESTVQTWFKPLAYAFILTRKSGYPHVFSGDMYGTKRNSPPEILALKHKIEPILKAR
**amyBL159**	**GQSLESTVQTWFKPLAYAFILTRESGYPQVFYGDMYGTKGDSQREIPALKHKIEPILKAR**
ABW90124.1	GQSLESTVQTWFKPLAYAFILTRESGYPQVFYGDMYGTKGDSQREIPALKHKIEPILKAR
ACN88151.1	GQSLESTVQTWFKPLAYAFILTRESGYPQVFYGDMYGTKGDSQREIPALKHKIEPILKAR
1BLI_A	GQSLESTVQTWFKPLAYAFILTRESGYPQVFYGDMYGTKGDSQREIPALKHKIEPILKAR
1OB0_A	GQSLESTVQTWFKPLAYAFILTRESGYPQVFYGDMYGTKGDSQREIPALKHKIEPILKAR
ABW94981.1	GQSLESTVQTWFKPLAYAFILTRESGYPQVFYGDMWGTKGDSQREIPALKHKIEPILKAR
1VJS_A	GQSLESTVQTWFKPLAYAFILTRESGYPQVFYGDMYGTKGDSQREIPALKHKIEPILKAR

```
6TOY_A          GQSLESTVQTWFKPLAYAFILTREAGYPQIFYGDMYGTKGASQREIPALKHKIEPILKAR
                ***********************::***::* ***:***  *  ** *************

WP_268400763.1  KQYAYGAQHDYFDHHDIVGWTREGDSSVANSGLAALITDGPGGAKRMYVGRQNAGETWHD
AMJ27400.1      KQYAYGAQHDYFDHHDIVGWTREGDSSVANSGLAALITDGPGGAKRMYVGRQNAGETWHD
amyBL159        KPYAYGAQHDYFDHHDIVGWTREGDSSVANSGLAALITDGPGGAKRMYVGRQNAGETWHD
ABW90124.1      KQYAYGAQHDYFDHHDIVGWTREGDSSVANSGLAALITDGPGGAKRMYVGRQNAGETWHD
ACN88151.1      KQYAYGAQHDYFDHHDIVGWTREGDSSVANSGLAALITDGPGGAKRMYVGRQNAGETWHD
1BLI_A          KQYAYGAQHDYFDHHDIVGWTREGDSSVANSGLAALITDGPGGAKRMYVGRQNAGETWHD
1OB0_A          KQYAYGAQHDYFDHHDIVGWTREGDSSVANSGLAALITDGPGGAKRMYVGRQNAGETWHD
ABW94981.1      KQYAYGAQHDYFDHHDIVGWTREGDSSVANSGLAALITDGPGGAKRMYVGRQNAGETWHD
1VJS_A          KQYAYGAQHDYFDHHDIVGWTREGDSSVANSGLAALITDGPGGAKRMYVGRQNAGETWHD
6TOY_A          KQYAYGAQHDYFDHHNIVGWTREGDSSVANSGLAALITDGPGGTKRMYVGRQNAGETWHD
                * *************:***************************:****************

WP_268400763.1  ITGNRSEPVVINSEGWGEFHVNGGSVSIYVQR
AMJ27400.1      ITGNRSEPVVI---------------------
amyBL159        ITGNRSEPVVINSEGWGEFHVNGGSVSIYVQR
ABW90124.1      ITGNRSEPVVINSEGWGEFHVNGGSVSIYVQR
ACN88151.1      ITGNRSEPVVINSEGWGEFHVNGGSVSIYVQR
1BLI_A          ITGNRSEPVVINSAGWGEFHVNGGSVSIYVQR
1OB0_A          ITGNRSEPVVINSEGWGEFHVNGGSVSIYVQR
ABW94981.1      ITGNRSEPVVIYSEGWGEFHVNGGSVSIYVQR
1VJS_A          ITGNRSEPVVINSEGWGEFHVNGGSVSIYVQR
6TOY_A          ITGNRSDSVVINAEGWGEFHVNGGSVSIYVQR
                ******:.***
```
